# Supplementary material for: Distinct Polymorphisms in HLA Class I Molecules Govern Their Susceptibility to Peptide Editing by TAPBPR
Source: Cell Rep. 2019 Nov 5;29(6):1621–1632.e3. doi: 10.1016/j.celrep.2019.09.074 (PMC7057265; doi:10.1016/j.celrep.2019.09.074)
Supplement: Document S1. Figures S1–S6 and Tables S1–S3 [file mmc1.pdf]

Cell Reports, Volume *29*

Supplemental Information

Distinct Polymorphisms in HLA Class I Molecules Govern Their Susceptibility to Peptide Editing by TAPBPR

F. Tudor Ilca, Linnea Z. Drexhage, Gemma Brewin, Sarah Peacock, and Louise H. Boyle

Supplementary information

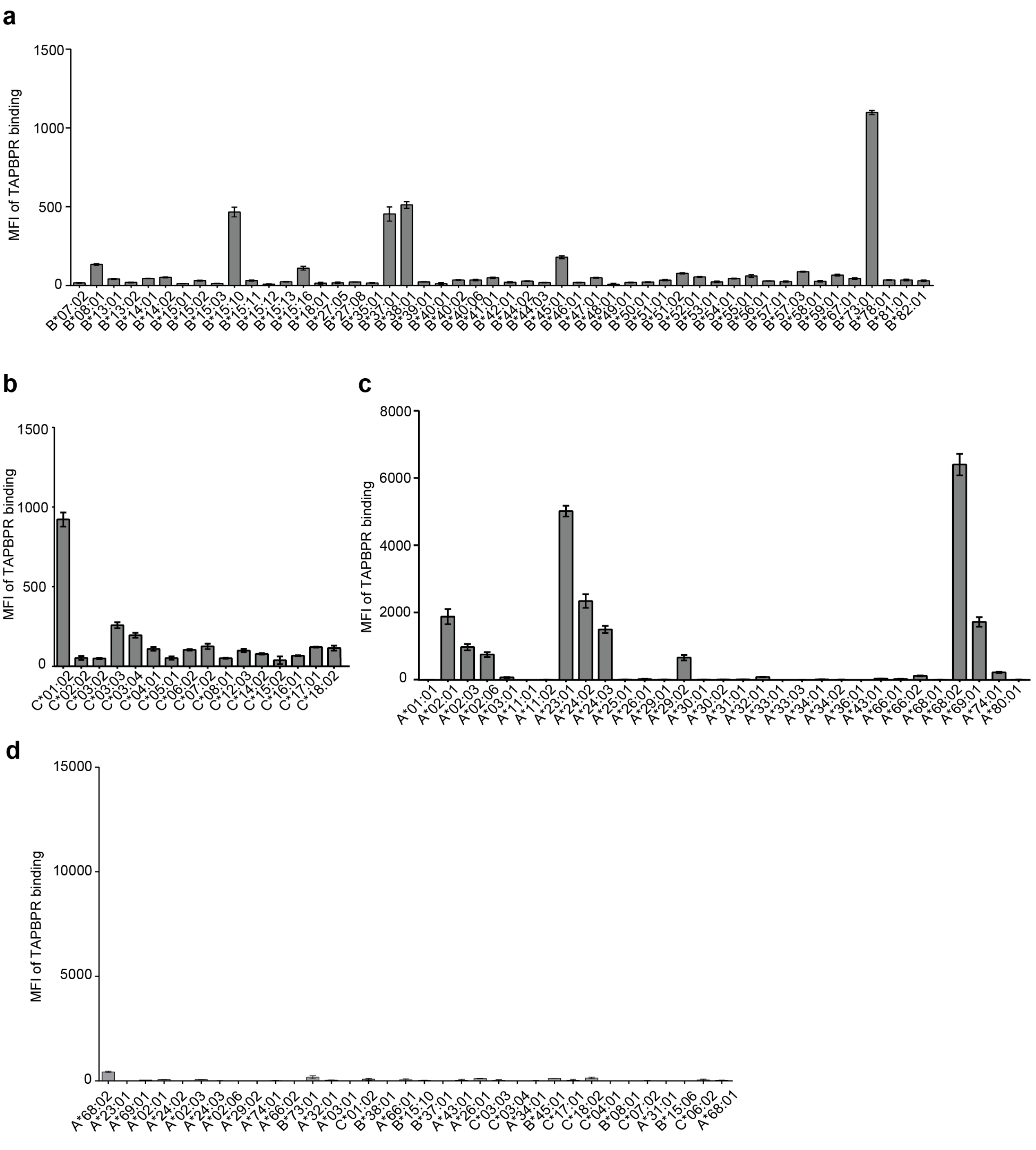

Figure S1. TAPBPR binding to HLA-B and -C molecules on the single HLA beads, Related to Figure 1. Bar graphs showing soluble TAPBPR binding to (**a**) HLA-B and (**b**) HLA-C molecules, upon treatment with 1 μM TAPBPR, to (**c**) HLA-A molecules treated with 100 nM TAPBPR and (**d**) with 1μM TN5 TAPBPR mutant to the top 34 HLA I binders to WT TAPBPR, as shown in **figure 1b**, using the SAB library. The SABs were treated with TAPBPR for 1 h at 22°C. The data including error bars was generated based on triplicates within one experiment. This is a representative example of three independent experiments.

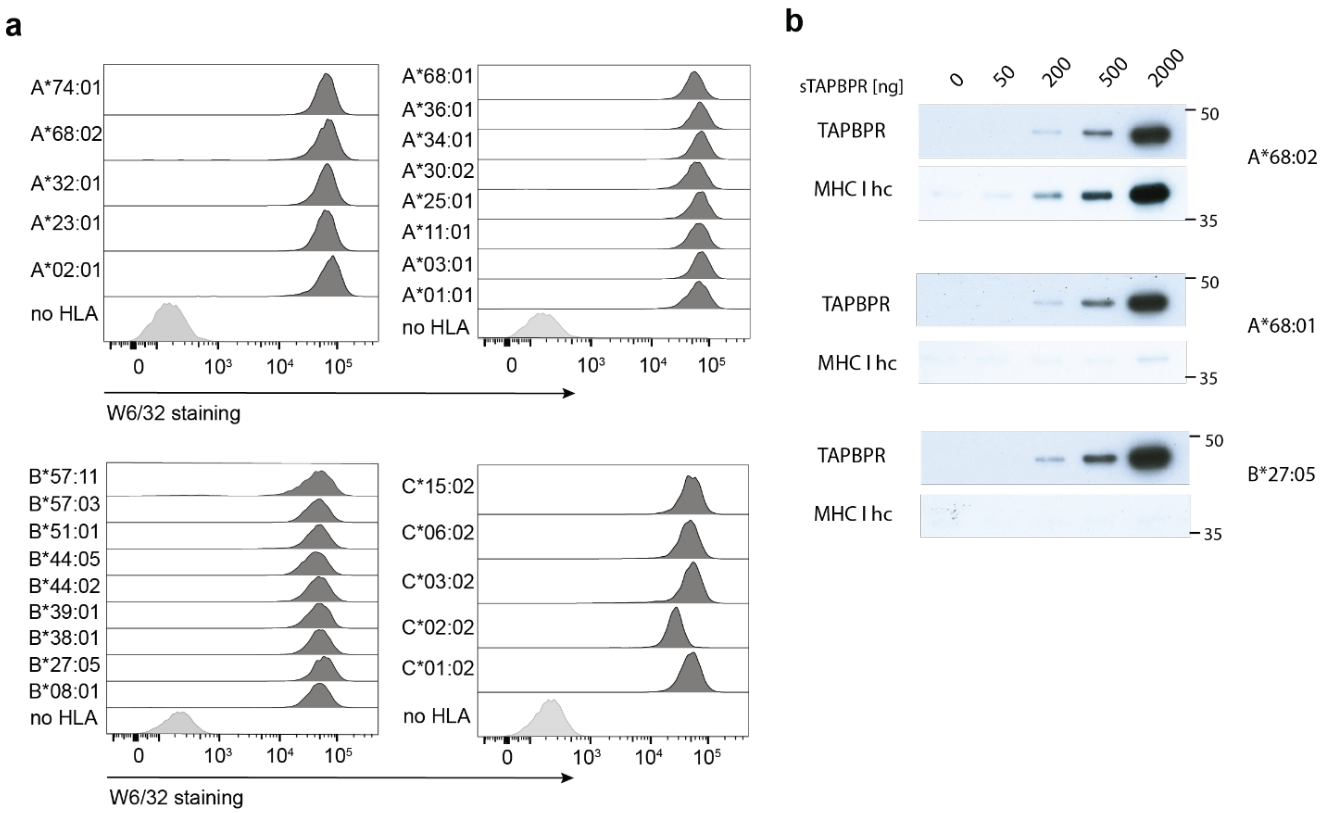

Figure S2. Levels of cell surface MHC class I molecules for individual HLA I alleles, Related to Figure 2 and Figure 3. (**a**) Histograms showing MHC class I levels detected with W6/32 antibody for each HLA I molecule transduced into HeLaM-HLA-ABC^{KO} cells (dark grey filled histograms). A light grey filled histogram was included for non-transduced HeLaM-HLA-ABC^{KO} cells. This is a representative example of three independent experiments. (**b**) Western blot analysis on recombinant TAPBPR pull-downs, when recombinant TAPBPR was titrated, on cells expressing A*68:02, A*68:01 or B*27:05. Membranes were probed for MHC I heavy chain (using HC10) and TAPBPR, as indicated. This is a representative experiment of two independent repeats.

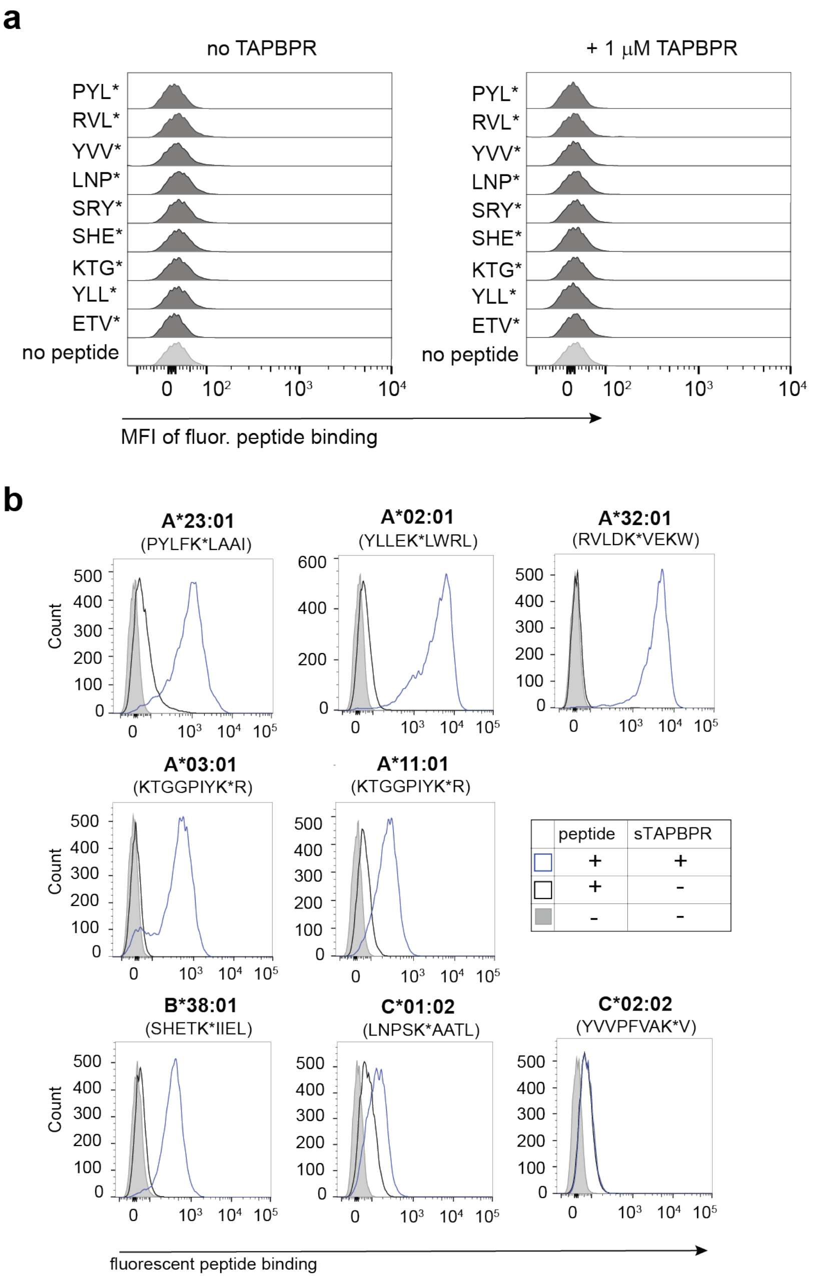

Figure S3. None of the peptides bind to HeLaM cells lacking classical HLA I molecules, Related to Figure 4. (**a)** Histograms showing binding levels of the fluorescent peptides used in **figure 4c** to HeLaM-HLA-ABC^{KO} cells, in the presence (right) or absence (left) of 1 μM TAPBPR. Cells were treated with TAPBPR for 15 min at 37°C, then peptide was added for 1 h at 37°C. The peptides tested were: ETVSK*QSNV (ETV*), YLLEK*LWRL (YLL*), KTGGPIYK*R (KTG*), SHETK*IIEL (SHE*), SRYWK*IRTR (SRY*), LNPSK*AATL (LNP*), YVVPFVAK*V (YVV*), RVLDK*VEKW (RVL*) and PYLFK*LAAI (PYL*). (**b**) Histograms showing the level of fluorescent peptide bound to individual HLA I-expressing cells, additional to the ones depicted in **figure 4b**, either untreated (filled grey line), treated with peptide alone (black line) or with peptide and TAPBPR (blue line). These histograms are representative of three independent experiments.

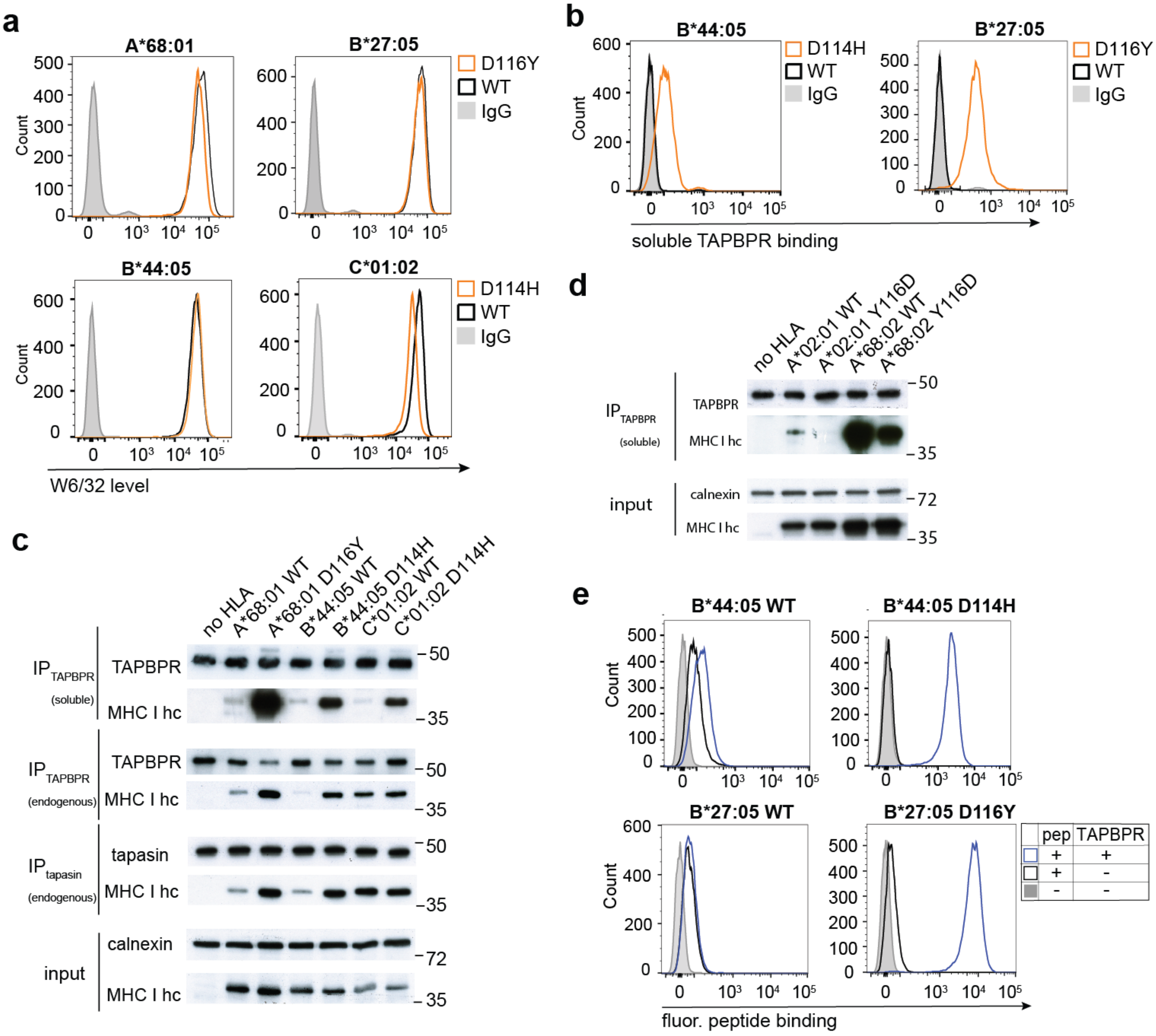

Figure S4. Disrupting the F pocket architecture impairs HLA I binding to TAPBPR, Related to Figure 6. (**a**) Histograms showing surface expression levels of A*68:01, B*27:05, B*44:05 and C*01:02 (black lines), compared to their corresponding F pocket mutants (orange lines). (**b**) Histograms showing the level of bound TAPBPR to cell surface B*44:05 or B*27:05 (black lines), as well as to their corresponding F pocket mutants (orange lines). Untreated cells are included as a negative control (solid grey line). (**c**) Western blot analysis on recombinant TAPBPR pull-downs (top) and endogenous TAPBPR and tapasin immunoprecipitates (bottom), on cells expressing A*68:01^{WT}, A*68:01^{D116Y}, B*44:05^{WT}, B*44:05^{D114H}, C*01:02^{WT} and C*01:02^{D114H} and on (**d**) recombinant TAPBPR pull-downs on cells expressing A*02:01, A*68:02, as well as their Y116D mutants. Membranes were probed for MHC I heavy chain, TAPBPR, tapasin, and calnexin, as indicated. (**e**) Histograms showing the level of bound fluorescent peptides (top) EFGK*AFSF to cells expressing either B*44:05^{WT} or B*44:05^{D114H}, and (bottom) SRYWK*IRTR to cells expressing either B*27:05^{WT} or B*27:05^{D116Y}, when cells were incubated with peptide alone (black line) or with peptide and 1 µM TAPBPR (blue line); an untreated sample was included as a negative control (solid grey line).

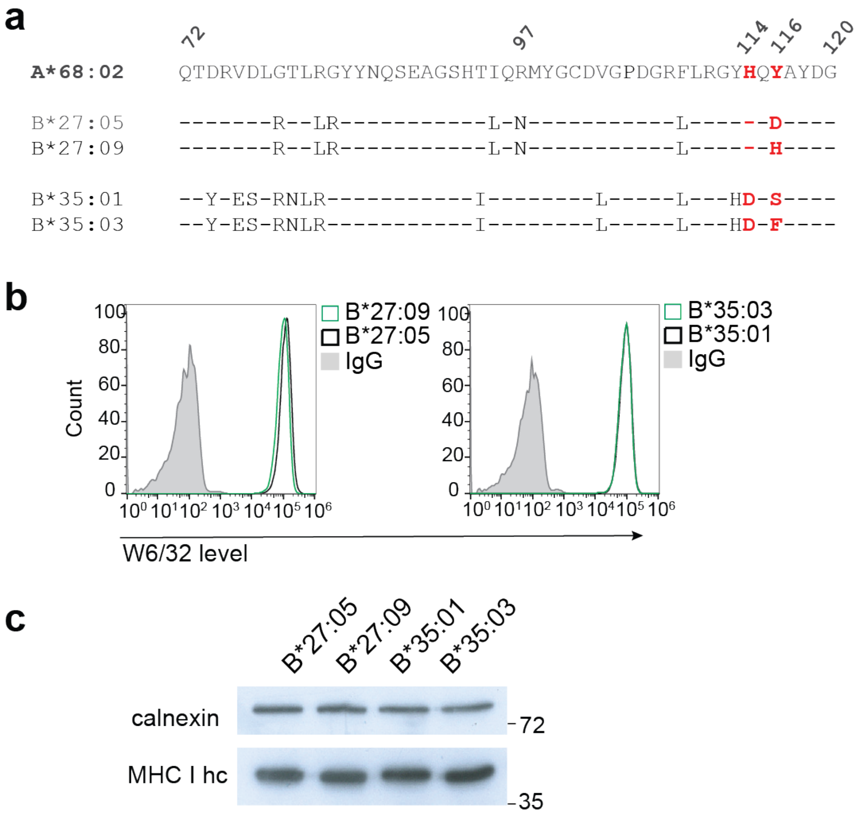

Figure S5. Expression of HLA-B molecules with natural polymorphisms at residue 116, Related to Figure 6. (**a**) Amino acid sequence alignment comparing residues 72-120 of A*68:02 with the HLA I pairs B*27:05 - B*27:09 and B*35:01 - B*35:03; residues 114 and 116 are highlighted in red. (**b**) Histograms showing the surface expression levels of the HLA-B molecules listed in (a), detected with W6/32 antibody. (**c**) Western blot analysis of the expression levels of HLA I molecules in whole cell lysates (a). Membranes were probed for MHC I heavy chain and calnexin, as indicated. These experiments are representative examples of three independent repeats.

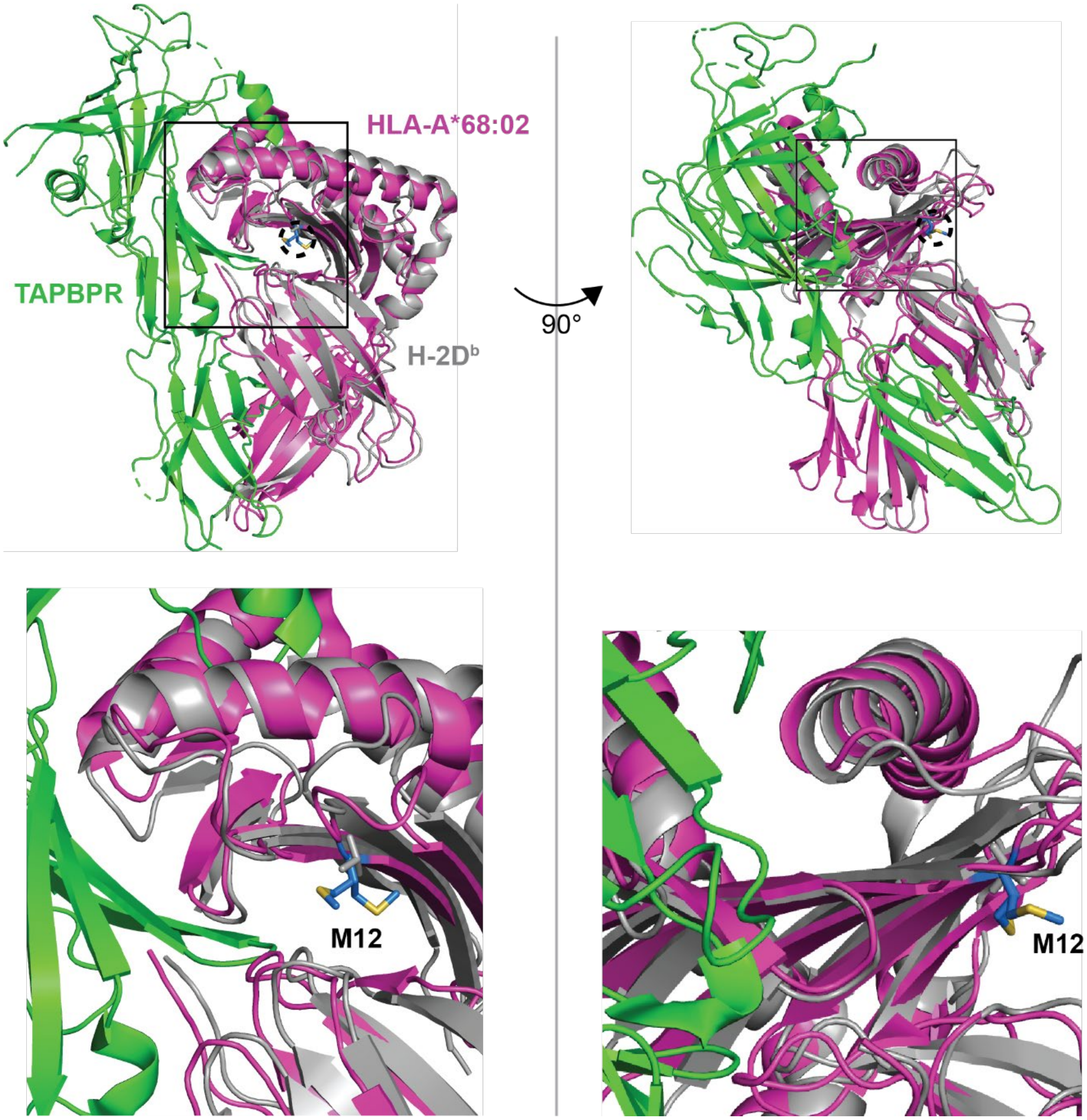

Figure S6. Residue M12 of HLA-A*68:02 does not appear to directly interact with TAPBPR, Related to Figure 7. PyMOL figure of the structure of HLA-A*68:02 folded with peptide SVYDFFVWL (pink) (PDB ID 4HX1) overlaid onto the structure of the H-2D^{b}:TAPBPR complex (grey and green respectively) (PDB ID 5OPI), depicted from different angles. Residue M12 of HLA-A*68:02 is colored in blue and highlighted in a dotted circle. Magnified selected areas are depicted below the whole structures. Residue M12 was captured in two different orientations in the crystal structure of HLA-A*68:02.

Table S1. MHC class I levels on single antigen HLA beads detected using W6/32, Related to Figure 1.

HLA allele	Bead #	MHC class I levels	HLA allele	Bead #	MHC class I levels	HLA allele	Bead #	MHC class I levels
A*01:01	3	18527	B*13:01	97	18064	B*51:02	66	18795
A*02:01	4	19739	B*13:02	36	19727	B*52:01	67	17497
A*02:03	5	18974	B*14:01	37	19502	B*53:01	68	18690
A*02:06	6	18702	B*14:02	38	17233	B*54:01	69	19465
A*03:01	7	19116	B*15:01	40	19608	B*55:01	70	19803
A*11:01	8	18910	B*15:02	41	19350	B*56:01	71	19119
A*11:02	9	19415	B*15:03	42	19668	B*57:01	72	18956
A*23:01	10	19552	B*15:10	43	20197	B*57:03	73	19594
A*24:02	11	19068	B*15:11	98	17639	B*58:01	74	18524
A*24:03	12	17692	B*15:12	44	19294	B*59:01	75	18090
A*25:01	13	19499	B*15:13	45	19296	B*67:01	76	19989
A*26:01	14	18366	B*15:16	46	18461	B*73:01	77	19542
A*29:01	15	19014	B*18:01	47	19915	B*78:01	78	17716
A*29:02	17	18142	B*27:05	16	19969	B*81:01	79	18679
A*30:01	18	17818	B*27:08	48	19129	B*82:01	80	17708
A*30:02	19	17921	B*35:01	49	19066	C*01:02	81	18988
A*31:01	20	18716	B*37:01	50	18551	C*02:02	82	16840
A*32:01	21	19548	B*38:01	51	18845	C*03:02	83	18982
A*33:01	22	18132	B*39:01	52	20136	C*03:03	84	19000
A*33:03	100	18062	B*40:01	53	19119	C*03:04	85	18943
A*34:01	23	19320	B*40:02	54	19336	C*04:01	86	12491
A*34:02	24	19845	B*40:06	99	18638	C*05:01	87	19122
A*36:01	25	17031	B*41:01	55	20023	C*06:02	88	16573
A*43:01	26	18328	B*42:01	56	20116	C*07:02	89	18133
A*66:01	27	18555	B*44:02	57	17659	C*08:01	90	19692
A*66:02	28	19088	B*44:03	58	18984	C*12:03	91	20447
A*68:01	29	19016	B*45:01	59	19214	C*14:02	92	17786
A*68:02	30	17506	B*46:01	63	19173	C*15:02	93	21276
A*69:01	31	18749	B*47:01	61	17077	C*16:01	94	14783
A*74:01	32	19216	B*48:01	62	19200	C*17:01	95	14369
A*80:01	33	18535	B*49:01	60	19506	C*18:02	96	18683
B*07:02	34	20419	B*50:01	64	19141			
B*08:01	35	20346	B*51:01	65	19516			

Table S2. Characterization of HLA-A molecules subjected to TAPBPR binding using the single antigen HLA beads, Related to Figure 5.

HLA allomorph	Supertype	F pocket specificity	Residue 114	Residue 116
**A*68:02**	**A2**	**Hydrophobic**	H	Y
**A*23:01**	**A24**	**Hydrophobic**	H	Y
**A*69:01**	**A2**	**Hydrophobic**	H	Y
**A*02:01**	**A2**	**Hydrophobic**	H	Y
**A*24:02**	**A24**	**Hydrophobic**	H	Y
**A*02:06**	**A2**	**Hydrophobic**	H	Y
A*03:01	A3	Basic	R	D
A*01:01	A1	Aromatic	R	D
A*26:01	A1	Aromatic	Q	D
A*30:01	A3	Basic / Aromatic	E	H
A*30:02	A1	Aromatic	E	H
A*33:01	A3	Basic	Q	D
A*36:01	A3	Basic	R	D
A*66:01	A3	Basic	Q	D
A*68:01	A3	Basic	R	D

- strong TAPBPR binders are highlighted in bold

Table S3: Primers used for cloning the MHC class I mutants, Related to STAR Methods.

Primer name	Sequence 5'-3'
B2705_D116Y_Fwd	GTACCACCAGTACGCCTACG
B2705_D116Y_Rev	CGTAGGCGTACTGGTGGTAC
B4405_D114H_Fwd	CGCGGGTATCATCAGTACGC
B4405_D114H_Rev	GCGTACTGATGATACCCGCG
C0102_D114H_Fwd	GCGGGTATCACCAGTACGC
C0102_D114H_Rev	GCGTACTGGTGATACCCGC
A0201_V12M_Fwd	CACATCCATGTCCCGGCC
A0201_V12M_Rev	GGCCGGGACATGGATGTG
A0201_S105P_Fwd	GACGTGGGGCCGGACTGG
A0201_S105P_Rev	CCAGTCCGGCCCCACGTC
A6802_M12V_Fwd	CTACACTTCCGTGTCCCGGC
A6802_M12V_Rev	GCCGGGACACGGAAGTGTAG
A6802_P105S_Fwd	CGTGGGGTCGGACGGG
A6802_P105S_Rev	CCCGTCCGACCCCACG
