# Supplementary material for: Distinct Polymorphisms in HLA Class I Molecules Govern Their Susceptibility to Peptide Editing by TAPBPR
Source: Cell Rep. 2019 Nov 5;29(6):1621–1632.e3. doi: 10.1016/j.celrep.2019.09.074 (PMC7057265; doi:10.1016/j.celrep.2019.09.074)
Supplement: Document S2. Article plus Supplemental Information [file mmc2.pdf]

Article

Cell Reports

Distinct Polymorphisms in HLA Class I Molecules Govern Their Susceptibility to Peptide Editing by TAPBPR

Graphical Abstract

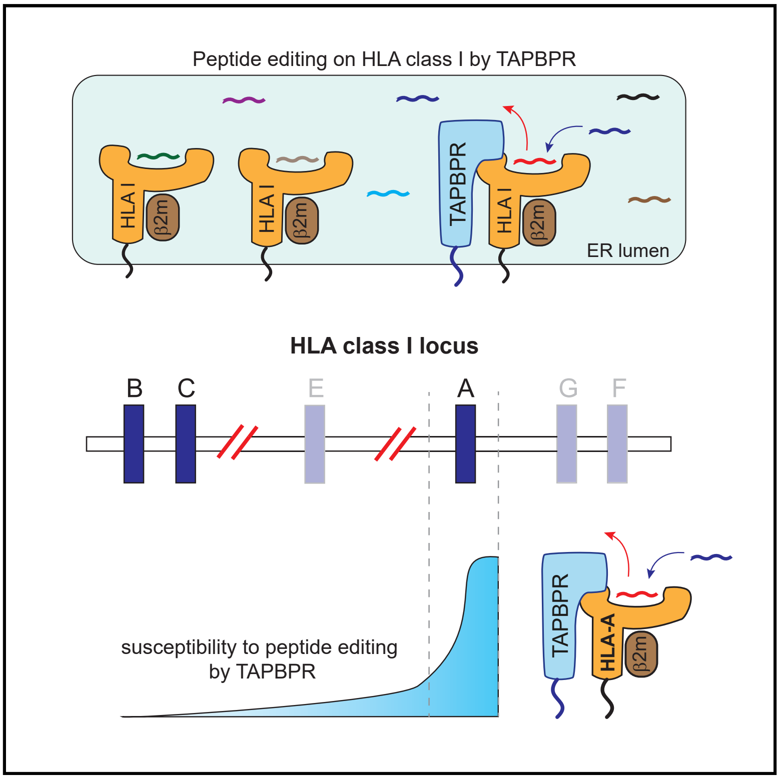

Authors

F. Tudor Ilca, Linnea Z. Drexhage, Gemma Brewin, Sarah Peacock, Louise H. Boyle

Correspondence

lhb22@cam.ac.uk

In Brief

Ilca et al. explore which human leukocyte antigen class I allotypes are subjected to TAPBPR-mediated peptide editing, revealing TAPBPR has preference for HLA-A, particularly for supertypes A2 and A24, over HLA-B and -C molecules.

Highlights

- TAPBPR exhibits a binding preference for HLA-A molecules over HLA-B and -C
- HLA-A2 and -A24 superfamily members are the strongest TAPBPR binders
- F pocket architecture impacts MHC I susceptibility to TAPBPR-mediated peptide editing

Ilca et al., 2019, Cell Reports *29*, 1621–1632
November 5, 2019 © 2019 The Author(s).
https://doi.org/10.1016/j.celrep.2019.09.074

OPEN ACCESS
CellPress

Distinct Polymorphisms in HLA Class I Molecules Govern Their Susceptibility to Peptide Editing by TAPBPR

F. Tudor Ilca,[1] Linnea Z. Drexhage,[2] Gemma Brewin,[3] Sarah Peacock,[3] and Louise H. Boyle[1,4,*]
[1]Department of Pathology, University of Cambridge, Tennis Court Road, Cambridge CB2 1QP, UK
[2]Faculty of Biology, University of Freiburg, Schaenzlestrasse 1, 79104 Freiburg, Germany
[3]Tissue Typing Laboratory, Box 209, Level 6 ATC, Cambridge University Hospitals, NHS Foundation Trust, Cambridge Biomedical Campus, Hills Road, Cambridge CB2 0QQ, UK
[4]Lead Contact
*Correspondence: lhb22@cam.ac.uk
https://doi.org/10.1016/j.celrep.2019.09.074

SUMMARY

Understanding how peptide selection is controlled on different major histocompatibility complex class I (MHC I) molecules is pivotal for determining how variations in these proteins influence our predisposition to infectious diseases, cancer, and autoinflammatory conditions. Although the intracellular chaperone TAPBPR edits MHC I peptides, it is unclear which allotypes are subjected to TAPBPR-mediated peptide editing. Here, we examine the ability of 97 different human leukocyte antigen (HLA) class I allotypes to interact with TAPBPR. We reveal a striking preference of TAPBPR for HLA-A, particularly for supertypes A2 and A24, over HLA-B and -C molecules. We demonstrate that the increased propensity of these HLA-A molecules to undergo TAPBPR-mediated peptide editing is determined by molecular features of the HLA-A F pocket, specifically residues H114 and Y116. This work reveals that specific polymorphisms in MHC I strongly influence their susceptibility to chaperone-mediated peptide editing, which may play a significant role in disease predisposition.

INTRODUCTION

Major histocompatibility complex class I (MHC I) molecules are transmembrane proteins that present fragments of the cellular proteome, in the form of short peptides, on the cell surface for inspection by cytotoxic T lymphocytes (CTLs). The MHC I locus contains the most polymorphic genes within humans, comprising over 10,000 different alleles. The vast majority of the polymorphisms in MHC I reside at the sites of the peptide binding groove that determine peptide specificity. Consequently, different MHC I molecules bind distinct sets of peptides and are, thus, capable of eliciting highly specific CD8$^+$ T cell responses. The peptide repertoire displayed by individual MHC I molecules has a critical influence on an individual's susceptibility to infectious diseases. For instance, specific pairs of MHC I molecules, such as HLA-B*35:01 and B*35:03, HLA-B*42:01 and B*42:02, and HLA-B*57:03 and B*57:02, that differ in only one amino acid are associated with different progression rates of HIV, mainly due to the different peptide repertoires presented (Gao et al., 2001; Kløverpris et al., 2012a, 2012b). Furthermore, although the inheritance of specific human leukocyte antigen (HLA) alleles, such as HLA-B*27:05 and HLA-B*51, is linked to certain autoinflammatory diseases (Fiorillo et al., 1998; Ohno et al., 1982), the role of these MHC I molecules in disease pathogenesis has yet to be fully elucidated. Thus, in-depth understanding regarding how polymorphisms in MHC I affect peptide selection, molecular stability, and their interactions with molecular chaperones is vital for understanding the role that variation in MHC I has on disease susceptibility.

Two molecular chaperones, tapasin and TAPBPR, play an important role in influencing the peptide repertoire presented on MHC I molecules to the immune system. Tapasin, the first discovered peptide editor for MHC I, works within the confines of the peptide-loading complex (PLC), which is the site where peptides are translocated from the cytoplasm into the endoplasmic reticulum (Ortmann et al., 1997; Sadasivan et al., 1996). Tapasin is responsible for loading peptide-receptive MHC I molecules with high-affinity peptides, by sequentially exchanging lower affinity peptides for higher affinity peptides (Chen and Bouvier, 2007; Howarth et al., 2004; Wearsch and Cresswell, 2007; Williams et al., 2002). Recent evidence suggests that TAPBPR functions as a second editor on the pathway, which, unlike tapasin, performs peptide exchange outside of the PLC (Boyle et al., 2013; Hermann et al., 2013, 2015; Morozov et al., 2016). Current data support a role for TAPBPR in refining the peptide repertoire displayed on MHC I (Hermann et al., 2015; Ilca et al., 2018a; Neerincx et al., 2017). Moreover, TAPBPR was shown to be capable of recruiting UDP-glucose:-glycoprotein glucosyltransferase 1 (UGT1) to reglucosylate MHC I and, consequently, recycle it to the PLC (Neerincx et al., 2017).

Although tapasin's ability to interact with and edit peptides on a wide variety of different HLA molecules, albeit mainly HLA-B allotypes (Rizvi et al., 2014; Williams et al., 2002; Peh et al., 1998), has been extensively investigated for over 20 years, knowledge regarding which HLA molecules TAPBPR is capable

This is an open access article under the CC BY license (http://creativecommons.org/licenses/by/4.0/).

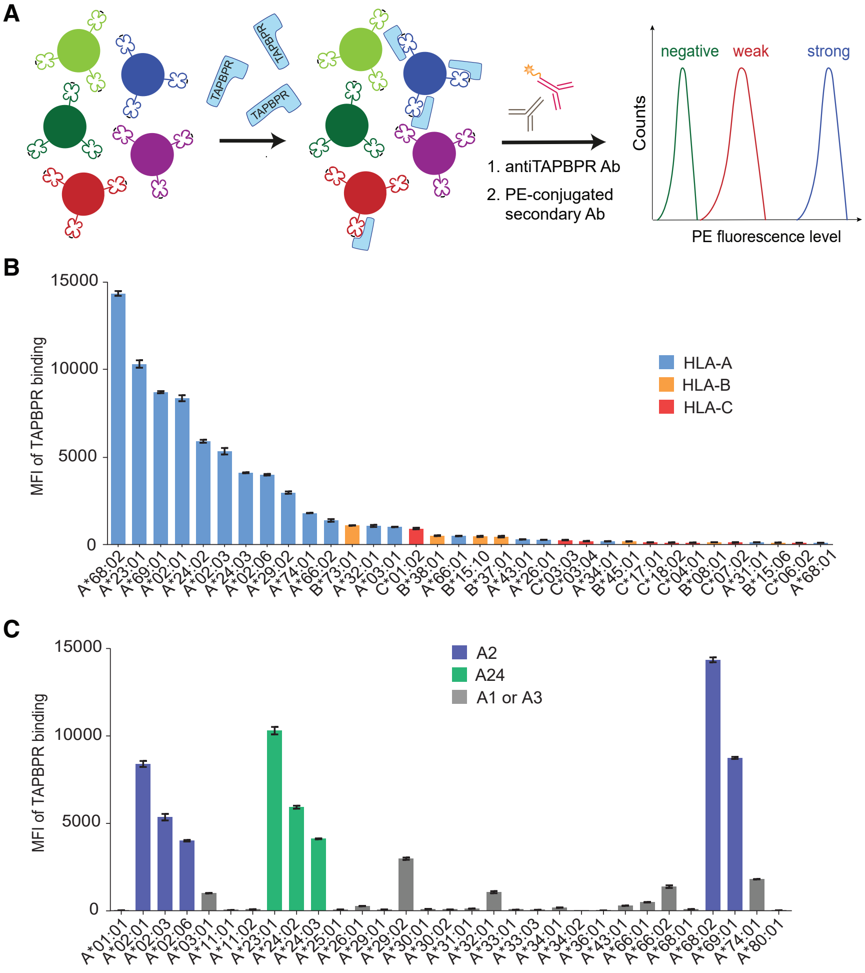

Figure 1. HLA-A Molecules, Particularly Members of the A2 and A24 Supertypes, Exhibit Stronger Binding to TAPBPR Than HLA-B and -C Molecules

(A) Schematic representation of the LABScreen SAB assay used to measure soluble TAPBPR binding to individual HLA I allotypes. The SABs were incubated with 1 μM TAPBPR or with 100 nM TAPBPR (Figure S1C) for 1 h, at 22°C, and then stained for TAPBPR.

(B) Bar graph showing the level of TAPBPR binding to the top 34 binders of the HLA I library, comprising HLA-A (blue), HLA-B (orange), and HLA-C (red) molecules.

(C) Bar graph summarizing TAPBPR binding to all HLA-A allotypes tested, with members of the HLA-A2 (blue) and -A24 (green) supertypes highlighted. TAPBPR binding to all HLA-B and -C allotypes tested is shown in Figures S1A and S1B, respectively.

Error bars show mean fluorescence intensity (MFI) ± SD from triplicates within one experiment. This experiment is representative of three independent experiments.

of performing peptide editing on is in its infancy by comparison (Hermann et al., 2015; Ilca et al., 2018a, 2018b; Morozov et al., 2016). Our recent work identifying a functional role of the TAPBPR K22-D35 loop in mediating peptide selection suggests that TAPBPR may have a prominent effect on some but not all MHC I molecules (Ilca et al., 2018a). Furthermore, variations in MHC I appear to influence the molecular mechanism by which TAPBPR shapes the peptide repertoire on MHC I (Ilca et al., 2018a). Thus, it is now crucial to understand on which HLA I molecules TAPBPR is able to function as a peptide editor.

Here, by comparing the ability of TAPBPR to bind to a panel of 97 different HLA I molecules, using LABScreen single antigen HLA class I beads (SABs), with further validation using cell-based assays, we reveal a striking preference of TAPBPR for HLA-A allotypes over HLA-B and -C and particularly for the members of the HLA-A2 and -A24 superfamilies. Furthermore, we identify that specific residues of the peptide-binding groove influence the propensity of MHC I to undergo TAPBPR-mediated peptide editing. This work reveals that MHC I polymorphisms strongly influence both chaperone association and, consequently, the level of peptide editing exerted on MHC I molecules.

RESULTS

HLA-A Molecules Exhibit Stronger Binding to TAPBPR Compared to HLA-B and -C Molecules

Although TAPBPR naturally functions on MHC I intracellularly, when given access to the plasma membrane, TAPBPR can bind to and exchange peptides on surface-expressed MHC I molecules (Ilca et al., 2018b). Thus far, we have only tested the ability of TAPBPR to bind to surface-expressed HLA-A2, HLA-A68, and H-2K^b (Ilca et al., 2018a, 2018b). Here, we sought to perform a comprehensive study of human TAPBPR binding to the products of different HLA I alleles in an unbiased manner. We compared the ability of soluble TAPBPR to bind to 97 different HLA I allotypes that are prevalent across different human subpopulations around the world by using SABs (Pei et al., 2003). The HLA I molecules coupled to these beads were produced and purified from Epstein Barr virus (EBV)-transformed cell lines and should, thus, be representative of the MHC I pool found in a cell, loaded with a wide range of peptides. Although, SABs are mainly used clinically to assess pre-transplant risks of allograft rejection (Wittenbrink et al., 2019), they have also provided insight into MHC I molecules:immune receptor interactions (Jones et al., 2011).

Here, SABs were treated with 1 μM soluble TAPBPR, and the levels of TAPBPR bound to each individual bead-coupled HLA I molecule were assessed using flow cytometry (Figure 1A). The screen revealed a striking preference of TAPBPR for HLA-A molecules over both HLA-B and -C allotypes (Figure 1B; Figure S1). Among all 97 allotypes screened, the top 11 strongest TAPBPR binders belong to the HLA-A group (Figure 1B). In addition, the top 4 strongest HLA-A binders, namely HLA-A*68:02, A*23:01,

OPEN ACCESS
CellPress

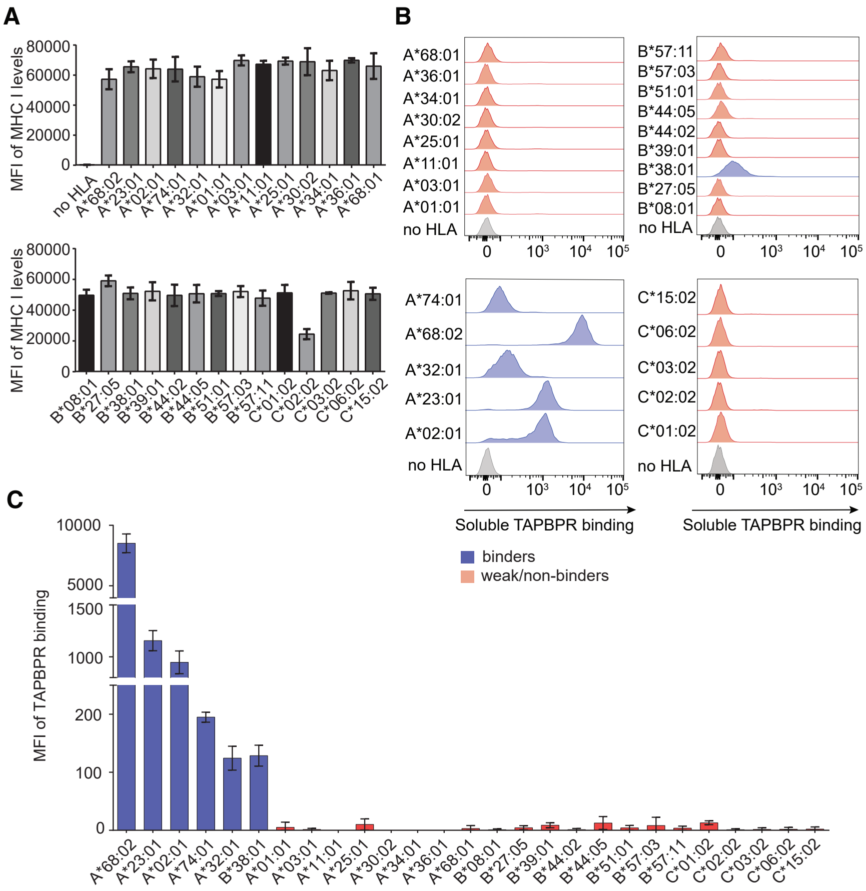

Figure 2. MHC I Molecules Show a Similar TAPBPR Binding Hierarchy in a Cellular System

(A) Bar graphs show surface expression levels of each HLA I allotype upon reconstitution in HeLaM-HLA-ABC^{KO} cells.

(B) Histograms depict levels of soluble TAPBPR bound to each HLA I allotype present at the cell surface, after cells were incubated with 1 μM TAPBPR for 30 min at 37°C; HLA I allotypes were grouped into either strong (blue) or weak (red) TAPBPR binders. HLA I-deficient cells were shown in gray.

(C) Bar graph summarizing TAPBPR binding measured as in (B).

Error bars show MFI ± SD from three independent experiments.

A*69:01, and A*02:01, all display >8-fold higher TAPBPR binding levels than the strongest binders among the HLA-B and -C groups, namely HLA-B*73:01 and HLA-C*01:02, respectively. In fact, the majority of HLA-B and -C allotypes on the SABs did not show significant binding to TAPBPR (Figures S1A and S1B). The staining of the SABs with W6/32 antibody revealed similar levels of peptide-loaded MHC I molecules across the HLA I library (Table S1). Moreover, the recombinant TN5 mutant of TAPBPR, which is unable to associate with MHC I (Hermann et al., 2013), showed no binding to the SABs (Figure S1D), demonstrating that TAPBPR does not associate non-specifically to the SABs.

HLA-A2 and -A24 Superfamily Members Are the Strongest TAPBPR Binders

Despite TAPBPR showing a clear preference for HLA-A over -B and -C allotypes, there were numerous HLA-A molecules on the SABs that did not show significant binding to TAPBPR, such as HLA-A*01:01, A*11:01, A*36:01, A*26:01, or A*68:01 (Figure 1C). Interestingly, the top eight strongest TAPBPR binders, namely A*68:02, A*23:01, A*69:01, A*02:01, A*24:02, A*02:03, A*24:03, and A*02:06, are all members of the HLA-A2 and -A24 superfamilies, according to previous classifications (Sidney et al., 2008), whereas the low binders are exclusively members of the HLA-A1 and -A3 supertypes. This classification of HLA I into supertypes was based on overlapping peptide repertoires, chemical specificity of both B and F pockets, and on the amino acid sequence similarity around the two pocket regions (Sidney et al., 2008).

HLA I Molecules Show a Similar TAPBPR-Binding Hierarchy in a Cellular System

Due to the limited information available regarding the precise composition of the HLA I molecules in the SABs, it was important to validate the results obtained from the SABs by using cellular systems. Recently, we have developed two cell-based assays to assess TAPBPR binding, as well as TAPBPR-mediated peptide exchange, on surface-expressed MHC I molecules (Ilca et al., 2018b). To test the ability of soluble TAPBPR to bind to different HLA I molecules expressed on cells, we first reconstituted HeLaM cells, in which HLA-A, -B, and -C heavy chains had been knocked out using CRISPR (HeLaM-HLA-ABC^{KO})(Neerincx and Boyle, 2019), with a panel of 27 individual HLA I allotypes that spanned the entire TAPBPR binding hierarchy (Figure 1). Flow cytometry using the monoclonal antibody (mAb) W6/32 showed similar surface expression levels for all 27 HLA I allotypes except HLA-C*02:02 (Figure 2A; Figure S2A).

We next assessed the ability of recombinant TAPBPR to bind to the surface of each cell line from the HeLaM HLA panel (Figures 2B and 2C). We observed no TAPBPR binding to HeLaM-HLA-ABC^{KO} cells (Figure 2B), as previously shown (Ilca et al., 2018b). The results showed a similar hierarchy of TAPBPR binding across MHC I expressed on the surface of cells compared to that found using the SABs (Figures 2B and 2C). The only exception was HLA-C*01:02, which showed low TAPBPR binding when present at the cell surface (Figures 2B and 2C). Consistent with the results obtained using the SABs, the top three strongest TAPBPR binders using our cellular system were HLA-A*68:02, A*23:01, and A*02:01 (Figures 2B and 2C). Strikingly, the binding of TAPBPR to HLA-A*68:02 was ~7.3- and ~9-fold higher than

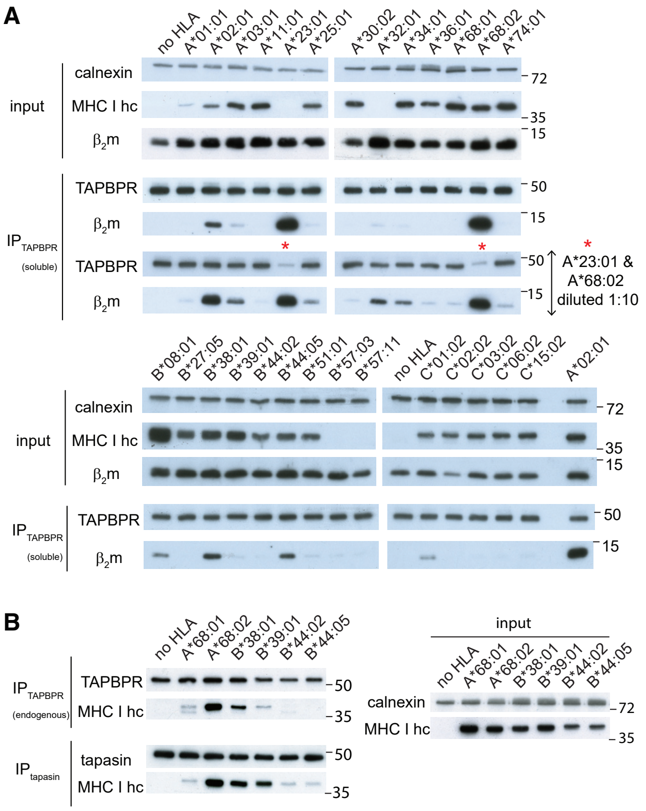

Figure 3. Intracellular Species of HLA I Molecules Confirm TAPBPR-Binding Hierarchy while Revealing Broader Reactivity to TAPBPR

Western blot analysis on (A) recombinant TAPBPR pull-downs from the lysate of each HLA I-reconstituted HeLaM-HLA-ABC^{KO} cell line and on (B) endogenous TAPBPR and tapasin immunoprecipitates. Membranes were probed with antibodies specific for MHC I heavy chain (using HC10 and HCA2), TAPBPR, tapasin, β_2m, and calnexin, as indicated. These are representative examples of three independent experiments.

to HLA-A*23:01 and A*02:01, respectively. HLA-A molecules belonging to the HLA-A1 and -A3 supertypes exhibited weak/no binding in comparison (Figures 2B and 2C). Thus, our cellular system confirms the clear preference of TAPBPR for members of the A2 and A24 HLA superfamilies. Among the HLA-B molecules tested, significant TAPBPR binding was only observed to HLA-B*38:01. No binding was observed to the other HLA-B molecules in the panel, including HLA-B*39:01 (Figures 2B and 2C), which is highly similar to HLA-B*38:01. Taken together, our results using surface-expressed MHC I molecules confirm that TAPBPR exhibits binding preference for MHC I molecules belonging to the HLA-A2 and -A24 superfamilies.

Intracellular Species of MHC I Molecules Confirm TAPBPR-Binding Hierarchy while Revealing Broader Reactivity to TAPBPR

Given that, naturally, TAPBPR is an intracellular MHC I chaperone, we next explored the ability of TAPBPR to bind to the total cellular pool of MHC I molecules by performing pull-down experiments by using recombinant TAPBPR on the whole-cell lysates of the panel of HeLaM cell lines expressing each of the 27 different MHC I allotypes (Figure 3A). Although flow cytometry confirmed the transduced MHC I molecules were well expressed (Figure 2A; Figure S2), some of the MHC I heavy chains were not reactive to either HC10 or HCA2 antibodies (Figure 3A). Therefore, the association of β_2m with TAPBPR was chosen as a surrogate readout for the TAPBPR:MHC I interaction. As expected, we did not observe an association between β_2m and TAPBPR in HeLaM-HLA-ABC^{KO} cells (Figure 3A), confirming the lack of β_2m pulled down directly onto TAPBPR. Using the pull-down system, we observed a similar TAPBPR binding hierarchy for MHC I molecules (Figure 3A) to that seen using both the SABs (Figure 1) and the cell surface binding assay (Figure 2). The pull-down experiments revealed that TAPBPR exhibited the strongest association with HLA-A*68:02 and A*23:01, followed by A*02:01 (Figure 3A), and that the association of TAPBPR with MHC I occurred in a dose-dependent manner (Figure S2B). Interestingly, when testing the binding of recombinant TAPBPR to the total cellular pool of HLA molecules, we now observed an interaction with some HLA allotypes of the A3 and A1 superfamilies (albeit significantly weaker than those observed with the HLA-A2 and -A24 superfamilies) that did not bind TAPBPR when present at the cell surface, such as A*03:01, A*68:01, A*34:01, and A*25:01 (Figure 3A). Among the HLA-B molecules tested, TAPBPR appeared to interact mainly with B*38:01, B*08:01, and B*44:05 (Figure 3A). Finally, HLA-C*01:02 was the only HLA-C molecule tested that showed binding to TAPBPR (Figure 3A). One likely reason for TAPBPR interacting with a significantly wider panel of MHC I molecules from whole-cell lysates compared to those on the plasma membrane is the availability of peptide-receptive MHC I or of MHC I molecules loaded with sub-optimal peptides. Given that the binding of TAPBPR and peptide to MHC I occurs in a competitive manner (Morozov et al., 2016; Thomas and Tampé, 2017; McShan et al., 2018), intracellular MHC I molecules, which contain a higher abundance of peptide-receptive molecules, are presumably more accessible to TAPBPR.

To verify that the relative binding of the different MHC I molecules to TAPBPR was not an artifact of using soluble recombinant TAPBPR, we next isolated endogenous TAPBPR by immunoprecipitation in several of the cell lines from the HeLaM HLA panel. The results were similar to the ones from the pull-down experiment performed with recombinant TAPBPR (Figure 3B). Namely, HLA-A*68:02 was a much stronger binder to TAPBPR than A*68:01, and binding of HLA-B*38:01 to TAPBPR was considerably stronger than that of B*39:01 (Figure 3B). However, HLA-B*44:05 exhibited very weak binding to endogenous TAPBPR (Figure 3B) compared to its binding observed to recombinant TAPBPR. Presumably, this result is due to the lower TAPBPR availability in the cell. These results suggest that MHC I molecules exhibit a wide affinity spectrum for TAPBPR, when considering natural expression levels of TAPBPR in an intracellular environment.

A Similar Hierarchy Is Observed for MHC I Binding to Tapasin

To shed further light on whether tapasin and TAPBPR work in synergy to shape the MHC I peptide repertoire, we explored whether the interactions of MHC I molecules with tapasin showed any correlation with the interactions observed with

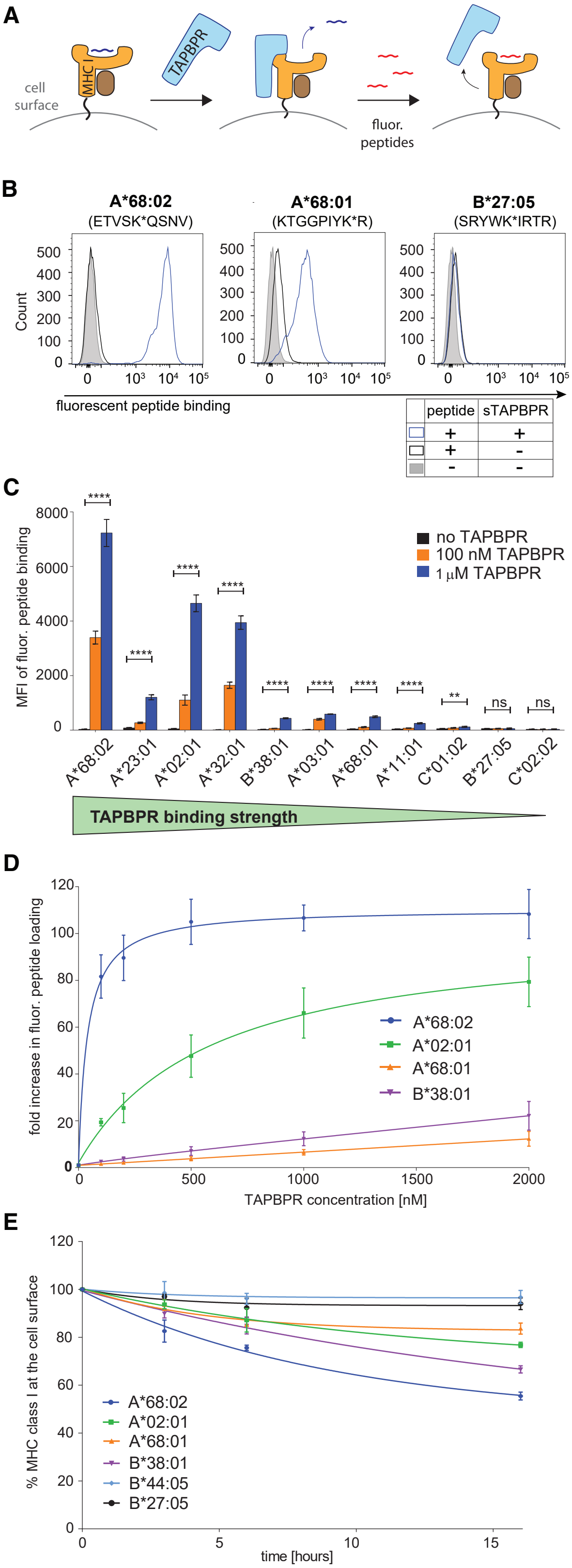

Figure 4. Peptide Exchange Exerted by TAPBPR Is Proportional to Its Ability to Bind HLA I

(A) Schematic representation of the TAPBPR-mediated peptide exchange assay on cell surface MHC I molecules. HeLaM-HLA-ABC^{KO} cells expressing individual HLA I allotypes were incubated with the corresponding fluorescently labeled peptides, in the presence of 1 μM TAPBPR.

(B) Histograms show levels of fluorescent peptide bound to HLA-A*68:02, A*68:01, and B*27:05, upon treatment without peptide (filled gray line), with peptide alone (black line) or with peptide and TAPBPR (blue line). Histograms depicting fluorescent peptide binding to the other HLA I allotypes tested are shown in Figure S3B.

(C) Bar graph summarizing fluorescent peptide binding to various HLA I in the presence of either no TAPBPR (black), 100 nM TAPBPR (orange), or 1 μM TAPBPR (blue).

(D) Line graph showing the fold increase in peptide binding to A*68:02, A*02:01, B*38:01, and A*68:01, by TAPBPR at different concentrations.

(E) BFA decay rates of the W6/32-reactive HLA I molecules listed in (D), as well as B*27:05 and B*44:05.

Error bars show MFI ± SD from three independent experiments. n/s, not significant; **$p \leq 0.01$, ****$p \leq 0.0001$, using unpaired two-tailed t test.

endogenous TAPBPR (Figure 3B). Immunoprecipitation of tapasin from the HeLaM cell panel expressing individual HLA molecules suggested that the HLA I allotypes that exhibited strong binding to TAPBPR were also strong binders to tapasin (Figure 3B). In the small panel tested, HLA-A*68:02 was the strongest binder to tapasin as well, whereas HLA-A*68:01 interacted weakly with tapasin (Figure 3B). Both B*44:05 and B*44:02 also interacted weakly with tapasin, as previously observed (Park et al., 2003), although these two HLA molecules exhibit different dependencies on tapasin (Rizvi et al., 2014; Williams et al., 2002). The strong interaction of HLA-A*68:02 with both tapasin and TAPBPR is in keeping with our previous finding that demonstrated that reglucosylation of this MHC I molecule by UGT1 associated with TAPBPR causes its recycling to the PLC complex (Neerincx et al., 2017).

The Peptide Exchange Activity of TAPBPR Is Proportional to Its Ability to Bind MHC I

Our recent work on a limited number of MHC I molecules suggests that the ability of soluble TAPBPR to bind to cell surface MHC I determines the efficiency of its peptide editing function (Ilca et al., 2018a). Therefore, we next tested the ability of TAPBPR to catalyze peptide exchange on a broad range of MHC I allotypes by using our recently developed peptide exchange assay on cell surface MHC I molecules (Ilca et al., 2018b) (Figure 4A). The MHC I allotypes selected for this experiment included 7 HLA-A, 2 HLA-B, and 2 HLA-C molecules, comprising strong TAPBPR binders (A*68:02, A*02:01, and A*23:01), weak TAPBPR binders (A*32:01, A*03:01, A*11:01, A*68:01, B*38:01, and C*01:02), and non-binders (B*27:05 and C*02:02). We designed fluorescently labeled peptides with high affinity for each of the 11 HLA I allotypes and measured the ability of the specific peptide to bind to HeLaM-HLA-ABC^{KO} cells reconstituted with the corresponding HLA I allotype by flow cytometry, in both the presence or absence of soluble TAPBPR (Figures 4 and S3).

In the absence of TAPBPR, very low levels of exogenous peptide binding were observed to all MHC I molecules (black line, Figures 4B and S3B). In the presence of TAPBPR, however,

peptide binding was significantly enhanced for the HLA allotypes classified as strong TAPBPR binders (blue line, Figures 4B, S3B, and summarized in 4C). The weak TAPBPR binders, namely A*03:01, A*11:01, A*68:01, B*38:01, and C*01:02 still showed a significant enhancement in peptide loading by TAPBPR (Figures 4B, S3B, and summarized in 4C); however, this enhancement was significantly lower than the one observed for the strong binders. Peptide loading on the TAPBPR non-binders, B*27:05 and C*02:02, was not enhanced in the presence of TAPBPR (Figures 4B, S3B, and 4C). None of the peptides tested showed any binding to HeLaM-HLA-ABC^{KO} cells (Figure S3A), confirming that the observed binding of each individual peptide occurred in an MHC I-dependent manner.

To better illustrate the differences in the peptide editing efficiency of TAPBPR across different MHC I allotypes, we measured the dose-dependent effect of TAPBPR on peptide exchange for both strong TAPBPR binders (A*68:02 and A*02:01) and weak binders (B*38:01 and A*68:01). Strikingly, the efficiency of TAPBPR-mediated peptide exchange was considerably higher on HLA-A*68:02 than on the other MHC I allotypes tested, including A*02:01 (Figure 4D). Namely, in the presence of 100 nM TAPBPR, an 80-fold increase in peptide loading on A*68:02 was observed compared to when peptide was added alone, a 20-fold increase was observed for A*02:01, whereas the low binders A*68:01 and B*38:01 only exhibited a <2.6-fold increase in peptide loading (Figure 4D). Moreover, TAPBPR reached half maximal effective concentration (EC_{50}) for peptide exchange at ~40 nM on A*68:02, whereas on A*02:01, the EC_{50} of TAPBPR was ~500 nM (Figure 4D). Taken together, these findings indicate a general correlation between the ability of TAPBPR to associate with MHC I molecules (Figures 1, 2, and 3) and the efficiency of its catalytic activity (Figure 4). The presence of outliers, such as HLA-A*32:01, is potentially due to specific intrinsic properties of particular MHC I allotypes that will allow TAPBPR to disrupt their binding groove and, thus, facilitate peptide dissociation to a high extent, without needing to form a strong interaction with the MHC I.

The Relative Susceptibility of MHC I Molecules to Undergo Peptide Editing by TAPBPR Does Not Correlate with Their Relative Stability at the Cell Surface

Given that each MHC I allotype will naturally present a distinct peptide repertoire, the stability of pMHC I complexes present at the cell surface may differ significantly among different MHC I allotypes. Thus, we next questioned whether the observed hierarchy regarding MHC I susceptibility to TAPBPR-mediated peptide exchange correlated with the relative stability of the MHC I molecules at the cell surface. By performing Brefeldin A (BFA) decay experiments on a few MHC I allotypes spanning the entire TAPBPR binding hierarchy (Figure 2C), we found that HLA-A*68:02, the strongest HLA I binder to TAPBPR, displayed the highest decay rate from the cell surface and, hence, the lowest stability among the allotypes tested (Figure 4E). In contrast, the weak TAPBPR binders B*44:05 and B*27:05 showed the highest relative stability among the molecules tested (Figure 4E). Based on this comparison alone, TAPBPR binding could appear to correlate with MHC I stability. However, highly similar decay rates were observed for A*02:01 and for A*68:01, which are at opposite ends of the TAPBPR binding hierarchy (Figure 4E). Moreover, HLA-B*38:01 showed a lower stability than A*02:01, despite its considerably weaker susceptibility to peptide editing by TAPBPR (Figure 4E). Overall, although the molecular stability of MHC I molecules may influence their propensity to undergo peptide editing by TAPBPR, this process appears to be driven mainly by the intrinsic ability of the MHC I to associate with TAPBPR.

Molecular Features of the F Pocket Correlate with MHC I Binding to TAPBPR

We have previously proposed that F pocket specificity for hydrophobic amino acids is a key requirement of MHC I molecules for undergoing efficient TAPBPR-mediated peptide exchange (Ilca et al., 2018a). Thus, we explored the molecular basis of the observed TAPBPR preference for the HLA-A2 and -A24 supertype members, focusing first on the MHC I F pocket. Our comparisons revealed that all members of the HLA-A2 and -A24 superfamilies exhibit F pocket specificity for hydrophobic amino acids (Table S2) (Sidney et al., 2008). In contrast, all members of the A3 superfamily accommodate a basic residue at this site (Table S2). Naturally, we next compared the residues involved in determining the F pocket specificity, found between positions 72 and 120 (Sidney et al., 2008), among different HLA-A molecules (Figure 5A). Interestingly, we found residues H114 and Y116 to be conserved across all members of the A2 and A24 superfamilies (Table S2; Figure 5A). Moreover, none of the other HLA I molecules currently known exhibit this combination of residues at the specified positions. Taken together, these correlations, based on a wide panel of HLA I allotypes, suggest that the susceptibility of HLA I molecules to TAPBPR-mediated peptide editing may be strongly influenced by the architecture of the MHC I F pocket (Ilca et al., 2018a).

The F Pocket Architecture Governs the Ability of MHC I Molecules to Associate with TAPBPR

We predicted that the combination of residues H114/Y116 was responsible for the strong interaction observed between MHC I and TAPBPR, by enabling an open conformation of the hydrophobic F pocket, accessible to the L30 residue of TAPBPR (Figure 5B). To test this, we artificially reconstituted these residues into several MHC I molecules. Although there are no HLA-B or -C allotypes that contain the H114/Y116 residue combination, HLA-B*27:05, for instance, contains H114 but D116 instead of Y116, and HLA-B*44:05 contains Y116 but D114 instead of H114 (Figure 6A). We, therefore, replaced D114 with a histidine in HLA-B*44:05 and replaced D116 with a tyrosine in HLA-B*27:05 (Figure 6A). Both HLA-B*44:05^{D114H} and B*27:05^{D116Y} showed very similar surface expression levels compared to their corresponding wild-type (WT) molecules when reconstituted into HeLaM-HLA-ABC^{KO} cells (Figure S4A).

When assessing their ability to bind TAPBPR, we found that neither B*27:05^{WT} nor B*44:05^{WT} associated significantly with TAPBPR at the cell surface (Figure 6B; Figure S4B). In stark contrast, both HLA-B*27:05^{D116Y} and B*44:05^{D114H} displayed a high level of TAPBPR binding (Figure 6B; Figure S4B), as was also observed when A*68:02-like F pocket was reconstituted into A*68:01 (Figure 6B), as previously shown (Ilca et al.,

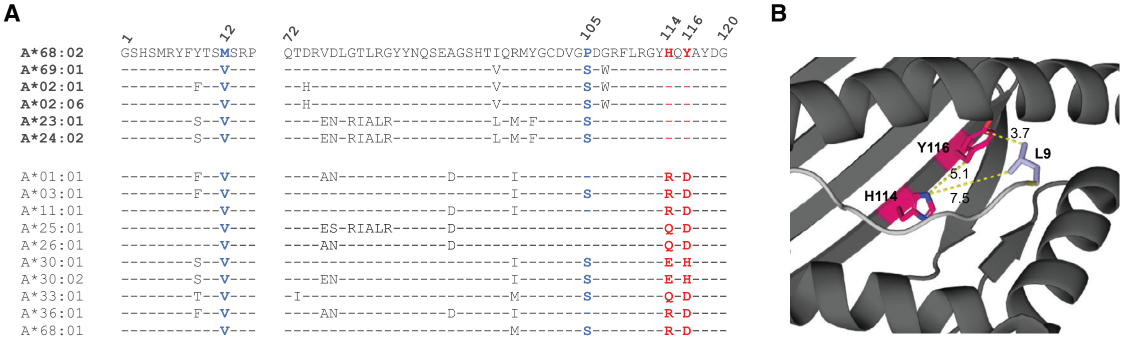

Figure 5. H114/Y116 Residue Combination Is Conserved Exclusively across All HLA I Members of the A2 and A24 Superfamilies

(A) Amino acid sequence alignment across members of all HLA-A superfamilies, spanning residues 1–12 and 72–120; residues 114 and 116 (red) as well as residues 12 and 105 (blue) were highlighted.

(B) PyMOL image of the structure of HLA-A*68:02 folded with peptide SVYDFFVWL (PDB ID 4HX1); residues H114 and Y116 are highlighted in red, the L9 residue of the peptide in light blue, and the peptide backbone in gray. Dotted yellow lines indicate the shortest distances between the connected amino acid residues, measured in Å.

2018a). TAPBPR pull-down experiments using whole-cell lysates verified these findings, while also demonstrating increased interaction between TAPBPR and HLA-C*01:02 upon introducing the H114/Y116 combination (Figure S4C). Moreover, disturbing the H114/Y116 F pocket combination of the strong TAPBPR binder A*68:02, by mutating Y116→D, triggered a severe decrease in TAPBPR binding (Figures 6B and S4D). These findings demonstrate that the H114/Y116 combination is a key determinant in the ability of HLA I molecules to interact with TAPBPR.

Interestingly, in addition to their stronger association with TAPBPR, HLA-A*68:01^{D116Y} and B*44:05^{D114H} showed a similar enhancement in their ability to bind tapasin compared to their WT counterparts (Figure S4C). This suggests there may be a correlation between the interaction of MHC I molecules with TAPBPR and their net binding to tapasin, similar to the one observed for the panel of WT HLA I molecules tested above (Figure 3B).

MHC I Susceptibility to TAPBPR-Mediated Peptide Editing Is Strongly Influenced by the F Pocket Architecture

We next explored whether the alterations made to the F pocket of HLA-B*44:05 and B*27:05 increased their susceptibility to TAPBPR-mediated peptide exchange. In the presence of TAPBPR, peptide binding to HLA-B*27:05 was not increased, whereas on HLA-B*44:05^{WT}, a slight enhancement was observed compared to peptide alone (Figures 6C and S4E). Strikingly, however, introducing an A*68:02-like F pocket in both HLA-B*44:05 and B*27:05 triggered a ~100-fold increase in peptide loading by TAPBPR (Figures 6C and S4E), to levels comparable to the ones observed for HLA-A*02:01 and A*68:02 molecules (Figure 4C). Upon testing whether the F pocket mutations altered the molecular stability of the MHC I molecules by using BFA decay assays, we observed that HLA-B*44:05^{D114H} displayed significantly lower stability at the cell surface than -B*44:05^{WT}, whereas B*27:05^{D116Y} was, surprisingly, more stable than B*27:05^{WT} (Figure 6D). Furthermore, mutating the F pocket of A*68:02 (A*68:02^{Y116D}) generated a slightly less stable molecule (Figure 6D), despite also severely impairing its ability to bind TAPBPR (Figure 6B). This further confirms that TAPBPR binding and peptide exchange on MHC I appear to be driven mainly by intrinsic properties of the MHC I rather than by the molecular stability of MHC I. Together, these findings indicate that residues H114 and Y116, in combination, represent a key molecular signature responsible for the high susceptibility observed for the HLA I members of the A2 and A24 supertypes to TAPBPR-mediated peptide exchange.

Natural Polymorphisms at Position 116 Influence the Propensity of HLA-B Molecules to Undergo TAPBPR-Mediated Peptide Editing

Having shown that artificially mutating residue 116 in HLA-B*27:05 dramatically increased it susceptibility to TAPBPR-mediated peptide editing, we next tested whether naturally occurring polymorphisms in HLA-B*27 at this position also influenced its ability to undergo peptide exchange. To this end, we assessed the ability of HLA-B*27:05 and B*27:09, which only differ in residue 116 (D→H) (Figure S5A) to undergo TAPBPR-mediated peptide exchange by testing their ability to bind to a fluorescent derivative of SRYWAIRTR (SRYWK*IRTR), which was shown to bind to both B*27:05 and B*27:09 (Nurzia et al., 2012). Although TAPBPR showed no significant effect on peptide binding to HLA-B*27:05, it facilitated an enhancement of over 100% in the level of peptide exchange on HLA-B*27:09 (Figure 6E). Next, we compared the ability of TAPBPR to mediate peptide exchange on another MHC I pair that also differs in residue 116 alone, namely HLA-B*35:01 and -B*35:03 (S116→F) (Figure S5A). Consequently, we tested the ability of TAPBPR to load a fluorescent derivative of SPAIFQSSM (SPAIK*QSSM), which was previously found to bind to both HLA-B35 molecules (HIV Molecular Immunology epitope database). Although the level of peptide exchange on HLA-B*35:01 was unaffected by TAPBPR, a slight (~20%) but significant increase in peptide loading onto B*35:03 was observed in the presence of TAPBPR (Figure 6E). As expected, given that none of these HLA-B

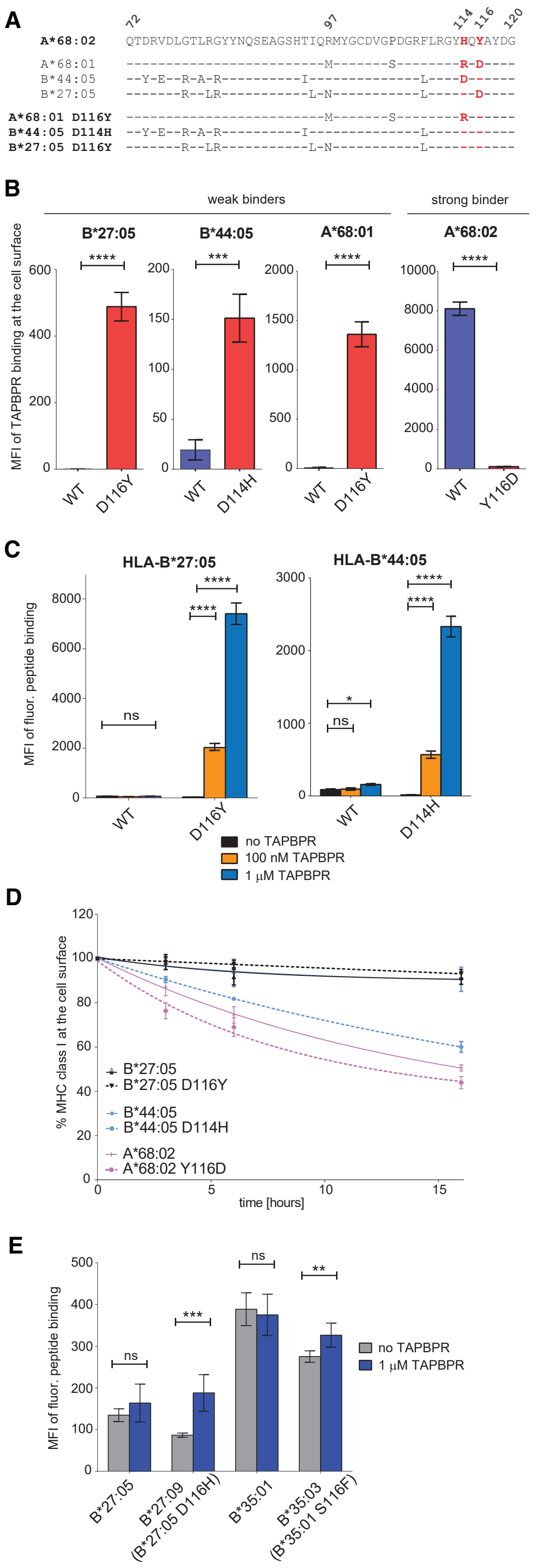

Figure 6. Residues H114/Y116 Promote Association of HLA-A, -B, and -C Molecules to TAPBPR

(A) Amino acid sequence alignment comparing residues 72–120 of A*68:02 with the ones of A*68:01, B*44:05, and B*27:05 and their corresponding F pocket mutants; residues 114 and 116 are highlighted in red.

(B) Bar graph shows TAPBPR binding to cells expressing each HLA I allotype in (A), either WT (blue) or mutant (red), based on three independent experiments.

(C) Bar graph shows the binding of fluorescent peptide SRYWK*IRTR to cells expressing either B*27:05^{WT} or B*27:05^{D116Y} and of EEFGK*AFSF to cells expressing either B*44:05^{WT} or B*44:05^{D114H} upon treatment with peptide and either no TAPBPR (black), 100 nM TAPBPR (orange), or 1 μM TAPBPR (blue).

(D) Line graph comparing the BFA decay rates of W6/32-reactive WT and mutated HLA I molecules.

(E) Bar graph showing the level of the fluorescent peptide SRYWK*IRTR to cell surface B*27:05 or B*27:09 and of SPAIK*QSSM to B*35:01 or B*35:03, when cells were treated either with peptide alone (gray bar) or peptide and 1 μM TAPBPR (blue bar). For expression levels of these HLA-I molecules, either at the cell surface or in the cell, see Figures S5B and S5C, respectively.

Error bars show MFI ± SD from three independent experiments. n/s, not significant; *p ≤ 0.05, **p ≤ 0.01, ***p ≤ 0.001, ****p ≤ 0.0001 using unpaired two-tailed t test.

molecules contain the H114/Y116 motif, the ability of TAPBPR to mediate peptide exchange on them was significantly lower compared to HLA-A2 and -A24 superfamily members. However, the one amino acid difference, at the key position 116, between the HLA-B*27 allotypes as well as between the HLA-B*35 allotypes, appears to be enough to cause a difference in the propensity of those molecules to undergo TAPBPR-mediated peptide editing.

M12 Residue, Present in HLA-A*68:02, Is Responsible for Its Distinct Ability to Interact with TAPBPR

Our data using both the SABs (Figure 1) and the cellular system (Figure 2) suggest that, although all members of the HLA-A2 and -A24 supertypes interact with TAPBPR, HLA-A*68:02 is by far the strongest TAPBPR binder. This implies that residues unique to HLA-A*68:02, apart from the ones found in the F pocket, enhance its ability to bind to TAPBPR. A comparison of the amino acid sequence of HLA-A*68:02 with the weak TAPBPR binder HLA-A*68:01 revealed that, in addition to residues determining the specificity of the F pocket (positions 97, 114, and 116), there are differences between these two MHC I allotypes at positions 12 and 105 (Figures 5A and 7A). Furthermore, among all members of the A2 and A24 superfamilies tested in this study, residues M12 and P105 were unique to HLA-A*68:02 (Figure 7A). For instance, HLA-A*69:01 shows a considerably lower ability to bind TAPBPR (Figure 1), although it differs from A*68:02 in only a few amino acids, including those at positions 12 and 105 (Figures 5A and 7A). We, therefore, hypothesized that residues M12 and/or P105 were responsible for the pronounced ability of HLA-A*68:02 to bind to TAPBPR. To test this, we swapped either residue 12 or 105 between HLA-A*68:02 and -A*02:01, resulting in the following mutants: A*68:02^{M12V}, A*68:02^{P105S}, A*02:01^{V12M}, and A*02:01^{S105P}. Upon reconstitution into HeLaM-HLA-ABC^{KO} cells, all four MHC I mutants showed similar surface expression as their WT counterparts (Figure 7B). When assessing the ability of these altered MHC I molecules to bind recombinant TAPBPR, we found that the mutation of residue 105 in either HLA-A*68:02 or

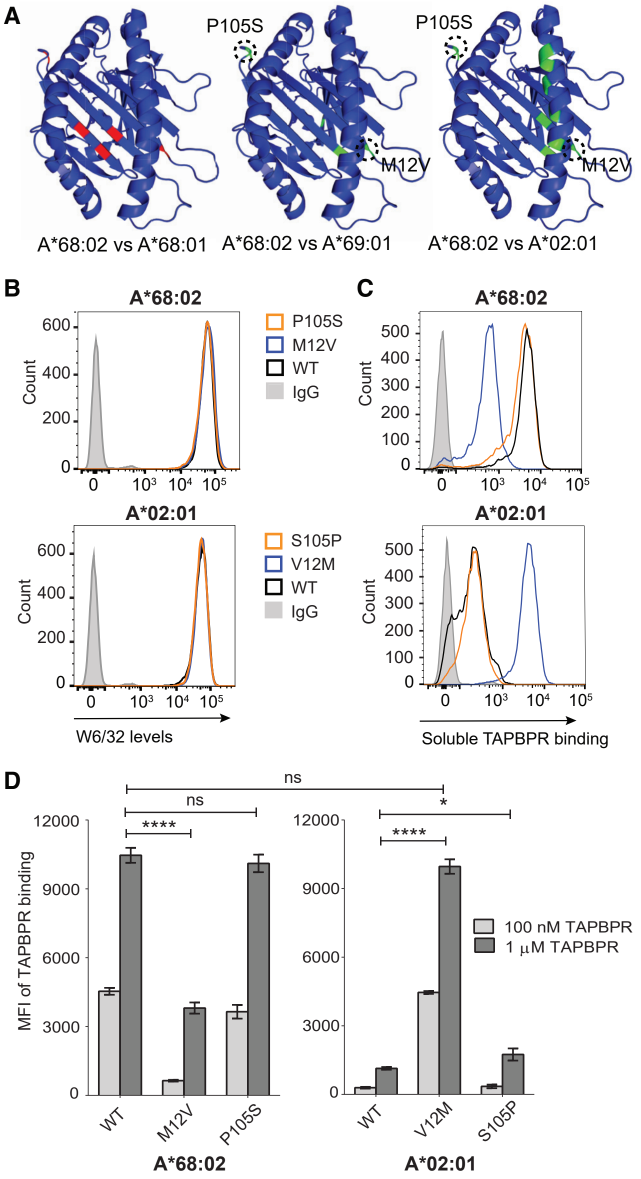

Figure 7. M12 Residue, Found Specifically in HLA-A*68:02, Promotes Its Accessibility to TAPBPR

(A) PyMOL figures highlighting amino acid differences between A*68:02 and A*68:01 (left), A*69:01 (center), and A*02:01 (right), respectively; conserved residues are colored in blue, different residues between A*68:02 and A*68:01 in red, and differences between A*68:02 and other strong binders in green.

(B) Histograms showing cell surface levels of A*68:02^{WT}, A*68:02^{M12V}, A*68:02^{P105S}, A*02:01^{WT}, A*02:01^{V12M}, and A*02:01^{S105P}.

(C) Histograms show TAPBPR binding to the HLA I variants in (B), after cells were treated with 100 nM TAPBPR for 30 min.

(D) Bar graphs showing TAPBPR binding to the HLA I allotypes tested in (C), upon treatment with either 100 nM (light gray) or 1 μM TAPBPR (dark gray). Bars show MFI ± SD from three independent experiments. n/s, not significant; *$p \leq 0.05$, ****$p \leq 0.0001$ using unpaired two-tailed t test.

A*02:01 had no significant effect on their ability to bind to TAPBPR (Figures 7C and 7D). Strikingly, however, the mutation of residue M12 in HLA-A*68:02 resulted in a 10-fold decrease in TAPBPR binding (Figures 7C and 7D). Furthermore, the corresponding V12M mutation in HLA-A*02:01 resulted in a >10-fold increase in TAPBPR binding (Figures 7C and 7D), to levels almost identical to those observed for A*68:02^{WT} (Figures 7C and 7D). These findings suggest that the M12 residue, uniquely present in HLA-A*68:02, is responsible for its distinct ability to interact with TAPBPR.

DISCUSSION

Here, we perform a large-scale study to explore which MHC I molecules interact with, and are consequently edited by, TAPBPR. We reveal a striking binding preference of TAPBPR for HLA-A allotypes, particularly for the members of the A2 and A24 superfamilies, over HLA-B and -C molecules. Our findings are in keeping with the limited number of HLA I molecules previously tested in TAPBPR binding and peptide exchange assays, namely HLA-A*02:01, A*68:02, B*08:01, and A*01:01 (Hermann et al., 2015; Ilca et al., 2018a, 2018b; Morozov et al., 2016).

Although the unique peptide repertoires bound to the various MHC I molecules may influence their molecular stability and, consequently, influence their propensity to undergo peptide editing by TAPBPR, our findings suggest that TAPBPR-mediated peptide editing is largely driven by the intrinsic ability of the MHC I to bind TAPBPR. Specifically, the molecular features of the MHC I F pocket appear to strongly influence the ability of MHC I to bind to TAPBPR. We show that the preference of TAPBPR for A2 and A24 HLA supertypes is due to their F pocket specificity for hydrophobic amino acid residues. Our findings are consistent with our previously proposed model that suggests that TAPBPR mediates efficient peptide exchange on MHC I by using its L30 residue to catalyze the dissociation of the C-terminal anchor residue of the peptide from the MHC I F pocket (Ilca et al., 2018a). Furthermore, our results establish that the residues found at positions 114 and 116 of MHC I are crucial for the observed allotype preference of TAPBPR for HLA I.

Interestingly, studies exploring tapasin function across a range of HLA I molecules, particularly HLA-B molecules, have previously proposed that residues 114 and 116 also affect MHC I dependency on tapasin (Park et al., 2003; Peh et al., 1998; Rizvi et al., 2014; Williams et al., 2002). Our results, based on a limited number of HLA I molecules, suggest that tapasin might display a similar HLA preference as TAPBPR. Although there appear to be shared features between tapasin and TAPBPR in terms of their MHC I preference and their proposed catalytic mechanisms (Fisette et al., 2016; Ilca et al., 2018a; McShan et al., 2018; Thomas and Tampé, 2017), there seems to be an obvious difference between the two peptide editors; although TAPBPR binding and peptide exchange is strongly dependent on F pocket specificity for hydrophobic amino acids, this does not appear to be the case for tapasin (Rizvi et al., 2014; Williams et al., 2002; Park et al., 2003). Moreover, tapasin and TAPBPR are likely to access distinct pools of MHC I in terms of glycan attachment (Neerincx and Boyle, 2019) and peptide affinity. Together, these findings indicate that the role of these two peptide editors on MHC I is likely to differ significantly. Although tapasin enables loading of peptide-receptive MHC I molecules, we speculate that TAPBPR plays a more significant role in dissociating peptides from MHC I

that have already gone through PLC-mediated peptide selection but are sub-optimal for presentation. To fulfil this purpose, in addition to the higher intrinsic affinity of TAPBPR for selected MHC I relative to tapasin, TAPBPR has developed a highly specialized loop region (Ilca et al., 2018a; Thomas and Tampé, 2017) that allows it to dissociate stably bound peptides, which are no longer accessible to tapasin. Consequently, TAPBPR has a stronger influence on MHC I molecules compatible with its functional region involved in peptide dissociation.

Apart from residues found in the F pocket, other polymorphisms in MHC I molecules will inevitably affect their susceptibility to TAPBPR. Here, we reveal that residue M12, found uniquely in HLA-A*68:02 among all HLA-A molecules tested, facilitates the accessibility of this allotype to TAPBPR. It is currently unclear how this subtle difference in one amino acid makes HLA-A*68:02 such a compatible ligand for TAPBPR. Modeling of this HLA-A molecule onto the solved crystal structure of human TAPBPR with mouse MHC I (Jiang et al., 2017; Thomas and Tampé, 2017) does not support a direct interaction between TAPBPR and MHC I at this site (Figure S6). It is possible that residue M12 affects the interaction between the heavy chain of A*68:02 and β_2m, potentially influencing the conformational plasticity of the MHC I complex (Figure S6), which was recently proposed to drive chaperone recognition (McShan et al., 2018).

In a biological context, our results suggest that HLA molecules accommodating basic/charged amino acids in their F pocket do not require extensive peptide editing by TAPBPR. In contrast, HLA-A molecules accommodating hydrophobic amino acids in this pocket appear to undergo significant peptide editing by TAPBPR. This could be due to both a wider availability of peptides carrying hydrophobic amino acids at their C terminus in the endoplasmic reticulum (ER) and an increased peptide-binding promiscuity of HLA-A2 and -A24 superfamily members, which would consequently require more stringent peptide selection. Given the preference of TAPBPR for HLA-A molecules over HLA-B and -C molecules, it is interesting to speculate whether rigorous peptide editing is required for MHC I molecules predominantly monitored by the T cell receptor, compared to those monitored by additional receptors on natural killer cells, such as the killer cell immunoglobulin-like receptors.

Interestingly, we have revealed that the subtle polymorphisms found in specific MHC I molecules associated with disease influence their ability to undergo TAPBPR-mediated peptide exchange. Although HLA-B*27:05 and -B*27:04 are strongly associated with ankylosing spondylitis (AS), HLA-B*27:06 and HLA-B*27:09 are either not or only weakly associated with AS. Here, we reveal that natural variations in residue 116 of these HLA-B27 molecules, which appear to correlate with disease susceptibility, impact their ability to undergo peptide editing by TAPBPR. Furthermore, we found that a difference in residue 116 between two MHC I molecules that are associated with different progression rates of HIV, HLA-B*35:01, and B*35:03, also influence their susceptibility to TAPBPR-mediated peptide editing. It is worth bearing in mind that our assays monitor the ability of TAPBPR to mediate peptide exchange on MHC I expressed on the cell surface, which have already passed through quality-control checkpoints. The magnitude of TAPBPR-mediated peptide editing on these specific MHC I molecules may be even greater when in its natural intracellular setting. Given that peptide selection likely plays an important role linking the association of specific MHC I molecules to infectious diseases and autoinflammatory conditions, our findings may offer new insight regarding how a apparently subtle variation in MHC I can have a significant impact on susceptibility to disease.

Regardless of its biological significance, the function of TAPBPR can be exploited to load immunogenic peptides onto surface-expressed MHC I molecules (Ilca et al., 2018b). Therefore, there may be a possibility to utilize recombinant TAPBPR for immunotherapeutic applications in the future, which may prove beneficial for increasing tumor immunogenicity. Our findings here suggest that a relatively broad range of HLA-A molecules, found at significant frequencies worldwide, are potentially targetable for exogenous peptide loading by human TAPBPR. The knowledge gained here may also prove informative regarding how to engineer TAPBPR to increase its compatibility and efficiency for an even wider range of MHC I molecules.

STAR★METHODS

Detailed methods are provided in the online version of this paper and include the following:

- KEY RESOURCES TABLE
- LEAD CONTACT AND MATERIALS AVAILABILITY
- EXPERIMENTAL MODEL AND SUBJECT DETAILS
- METHOD DETAILS
  - Constructs
  - Lentiviral transductions
  - Antibodies
  - MHC I-binding peptides
  - Expression and purification of TAPBPR protein
  - Flow cytometry
  - Peptide binding assays
  - BFA decay assays
  - Single antigen bead screen
  - Immunoprecipitation, gel electrophoresis and western blotting
- QUANTIFICATION AND STATISTICAL ANALYSIS
- DATA AND CODE AVAILABILITY

SUPPLEMENTAL INFORMATION

Supplemental Information can be found online at https://doi.org/10.1016/j.celrep.2019.09.074.

ACKNOWLEDGMENTS

We are grateful to Andreas Neerincx and Alison McDowall (University of Cambridge) for providing reagents essential for this work. We thank Ida Hafstrand, John Trowsdale, and James Traherne (University of Cambridge) for useful discussions. This work was funded by a Wellcome Senior Research Fellowship (104647/Z/14/Z) awarded to L.H.B. and a Wellcome PhD studentship (109076/Z/15/A) awarded to F.T.I.

AUTHOR CONTRIBUTIONS

F.T.I. conceived and designed the work; acquired, formally analyzed, and interpreted data; and wrote the manuscript. L.Z.D. acquired and analyzed

data. G.B. acquired and analyzed data. S.P. designed and supervised work. L.H.B. conceived and designed the work, interpreted data, generated novel resources, supervised work, and wrote and revised the manuscript.

DECLARATION OF INTERESTS

Part of the work described in this paper has been the subject of a patent application filed by Cambridge Enterprise, a wholly owned subsidiary of the University of Cambridge. The patent application number is PCT/EP2019/051907, with F.T.I. and L.H.B. as named inventors.

Received: March 11, 2019
Revised: July 28, 2019
Accepted: September 25, 2019
Published: November 5, 2019

REFERENCES

Barnstable, C.J., Bodmer, W.F., Brown, G., Galfre, G., Milstein, C., Williams, A.F., and Ziegler, A. (1978). Production of monoclonal antibodies to group A erythrocytes, HLA and other human cell surface antigens-new tools for genetic analysis. Cell *14*, 9–20.

Boyle, L.H., Hermann, C., Boname, J.M., Porter, K.M., Patel, P.A., Burr, M.L., Duncan, L.M., Harbour, M.E., Rhodes, D.A., Skjødt, K., et al. (2013). Tapasin-related protein TAPBPR is an additional component of the MHC class I presentation pathway. Proc. Natl. Acad. Sci. USA *110*, 3465–3470.

Chen, M., and Bouvier, M. (2007). Analysis of interactions in a tapasin/class I complex provides a mechanism for peptide selection. EMBO J. *26*, 1681–1690.

Dick, T.P., Bangia, N., Peaper, D.R., and Cresswell, P. (2002). Disulfide bond isomerization and the assembly of MHC class I-peptide complexes. Immunity *16*, 87–98.

Fiorillo, M.T., Greco, G., Maragno, M., Potolicchio, I., Monizio, A., Dupuis, M.L., and Sorrentino, R. (1998). The naturally occurring polymorphism Asp116–>His116, differentiating the ankylosing spondylitis-associated HLA-B*2705 from the non-associated HLA-B*2709 subtype, influences peptide-specific CD8 T cell recognition. Eur. J. Immunol. *28*, 2508–2516.

Fisette, O., Wingbermühle, S., Tampé, R., and Schäfer, L.V. (2016). Molecular mechanism of peptide editing in the tapasin-MHC I complex. Sci. Rep. *6*, 19085.

Gao, X., Nelson, G.W., Karacki, P., Martin, M.P., Phair, J., Kaslow, R., Goedert, J.J., Buchbinder, S., Hoots, K., Vlahov, D., et al. (2001). Effect of a single amino acid change in MHC class I molecules on the rate of progression to AIDS. N. Engl. J. Med. *344*, 1668–1675.

Hermann, C., Strittmatter, L.M., Deane, J.E., and Boyle, L.H. (2013). The binding of TAPBPR and Tapasin to MHC class I is mutually exclusive. J. Immunol. *191*, 5743–5750.

Hermann, C., van Hateren, A., Trautwein, N., Neerincx, A., Duriez, P.J., Stevanović, S., Trowsdale, J., Deane, J.E., Elliott, T., and Boyle, L.H. (2015). TAPBPR alters MHC class I peptide presentation by functioning as a peptide exchange catalyst. eLife *4*, e09617.

Howarth, M., Williams, A., Tolstrup, A.B., and Elliott, T. (2004). Tapasin enhances MHC class I peptide presentation according to peptide half-life. Proc. Natl. Acad. Sci. USA *101*, 11737–11742.

Ilca, F.T., Neerincx, A., Hermann, C., Marcu, A., Stevanović, S., Deane, J.E., and Boyle, L.H. (2018a). TAPBPR mediates peptide dissociation from MHC class I using a leucine lever. eLife *7*, e40126.

Ilca, F.T., Neerincx, A., Wills, M.R., de la Roche, M., and Boyle, L.H. (2018b). Utilizing TAPBPR to promote exogenous peptide loading onto cell surface MHC I molecules. Proc. Natl. Acad. Sci. USA *115*, E9353–E9361.

Jiang, J., Natarajan, K., Boyd, L.F., Morozov, G.I., Mage, M.G., and Margulies, D.H. (2017). Crystal structure of a TAPBPR-MHC I complex reveals the mechanism of peptide editing in antigen presentation. Science *358*, 1064–1068.

Jones, D.C., Kosmoliaptsis, V., Apps, R., Lapaque, N., Smith, I., Kono, A., Chang, C., Boyle, L.H., Taylor, C.J., Trowsdale, J., and Allen, R.L. (2011). HLA class I allelic sequence and conformation regulate leukocyte Ig-like receptor binding. J. Immunol. *186*, 2990–2997.

Kløverpris, H.N., Harndahl, M., Leslie, A.J., Carlson, J.M., Ismail, N., van der Stok, M., Huang, K.H., Chen, F., Riddell, L., Steyn, D., et al. (2012a). HIV control through a single nucleotide on the HLA-B locus. J. Virol. *86*, 11493–11500.

Kloverpris, H.N., Stryhn, A., Harndahl, M., van der Stok, M., Payne, R.P., Matthews, P.C., Chen, F., Riddell, L., Walker, B.D., Ndung'u, T., et al. (2012b). HLA-B*57 Micropolymorphism shapes HLA allele-specific epitope immunogenicity, selection pressure, and HIV immune control. J. Virol. *86*, 919–929.

McShan, A.C., Natarajan, K., Kumirov, V.K., Flores-Solis, D., Jiang, J., Badstübner, M., Toor, J.S., Bagshaw, C.R., Kovrigin, E.L., Margulies, D.H., and Sgourakis, N.G. (2018). Peptide exchange on MHC-I by TAPBPR is driven by a negative allostery release cycle. Nat. Chem. Biol. *14*, 811–820.

Morozov, G.I., Zhao, H., Mage, M.G., Boyd, L.F., Jiang, J., Dolan, M.A., Venna, R., Norcross, M.A., McMurtrey, C.P., Hildebrand, W., et al. (2016). Interaction of TAPBPR, a tapasin homolog, with MHC-I molecules promotes peptide editing. Proc. Natl. Acad. Sci. USA *113*, E1006–E1015.

Neerincx, A., and Boyle, L.H. (2019). Preferential interaction of MHC class I with TAPBPR in the absence of glycosylation. Mol. Immunol. *113*, 58–66.

Neerincx, A., Hermann, C., Antrobus, R., van Hateren, A., Cao, H., Trautwein, N., Stevanović, S., Elliott, T., Deane, J.E., and Boyle, L.H. (2017). TAPBPR bridges UDP-glucose:glycoprotein glucosyltransferase 1 onto MHC class I to provide quality control in the antigen presentation pathway. eLife *6*, e23049.

Nurzia, E., Narzi, D., Cauli, A., Mathieu, A., Tedeschi, V., Caristi, S., Sorrentino, R., Böckmann, R.A., and Fiorillo, M.T. (2012). Interaction pattern of Arg 62 in the A-pocket of differentially disease-associated HLA-B27 subtypes suggests distinct TCR binding modes. PLoS One *7*, e32865.

Ohno, S., Ohguchi, M., Hirose, S., Matsuda, H., Wakisaka, A., and Aizawa, M. (1982). Close association of HLA-Bw51 with Behçet's disease. Arch. Ophthalmol. *100*, 1455–1458.

Ortmann, B., Copeman, J., Lehner, P.J., Sadasivan, B., Herberg, J.A., Grandea, A.G., Riddell, S.R., Tampé, R., Spies, T., Trowsdale, J., and Cresswell, P. (1997). A critical role for tapasin in the assembly and function of multimeric MHC class I-TAP complexes. Science *277*, 1306–1309.

Park, B., Lee, S., Kim, E., and Ahn, K. (2003). A single polymorphic residue within the peptide-binding cleft of MHC class I molecules determines spectrum of tapasin dependence. J. Immunol. *170*, 961–968.

Peh, C.A., Burrows, S.R., Barnden, M., Khanna, R., Cresswell, P., Moss, D.J., and McCluskey, J. (1998). HLA-B27-restricted antigen presentation in the absence of tapasin reveals polymorphism in mechanisms of HLA class I peptide loading. Immunity *8*, 531–542.

Pei, R., Lee, J.H., Shih, N.J., Chen, M., and Terasaki, P.I. (2003). Single human leukocyte antigen flow cytometry beads for accurate identification of human leukocyte antigen antibody specificities. Transplantation *75*, 43–49.

Rizvi, S.M., Salam, N., Geng, J., Qi, Y., Bream, J.H., Duggal, P., Hussain, S.K., Martinson, J., Wolinsky, S.M., Carrington, M., and Raghavan, M. (2014). Distinct assembly profiles of HLA-B molecules. J. Immunol. *192*, 4967–4976.

Sadasivan, B., Lehner, P.J., Ortmann, B., Spies, T., and Cresswell, P. (1996). Roles for calreticulin and a novel glycoprotein, tapasin, in the interaction of MHC class I molecules with TAP. Immunity *5*, 103–114.

Sidney, J., Peters, B., Frahm, N., Brander, C., and Sette, A. (2008). HLA class I supertypes: a revised and updated classification. BMC Immunol. *9*, 1.

Stam, N.J., Spits, H., and Ploegh, H.L. (1986). Monoclonal antibodies raised against denatured HLA-B locus heavy chains permit biochemical characterization of certain HLA-C locus products. J. Immunol. *137*, 2299–2306.

Stam, N.J., Vroom, T.M., Peters, P.J., Pastoors, E.B., and Ploegh, H.L. (1990). HLA-A- and HLA-B-specific monoclonal antibodies reactive with free heavy

chains in western blots, in formalin-fixed, paraffin-embedded tissue sections and in cryo-immuno-electron microscopy. Int. Immunol. *2*, 113–125.

Thomas, C., and Tampé, R. (2017). Structure of the TAPBPR-MHC I complex defines the mechanism of peptide loading and editing. Science *358*, 1060–1064.

Wearsch, P.A., and Cresswell, P. (2007). Selective loading of high-affinity peptides onto major histocompatibility complex class I molecules by the tapasin-ERp57 heterodimer. Nat. Immunol. *8*, 873–881.

Williams, A.P., Peh, C.A., Purcell, A.W., McCluskey, J., and Elliott, T. (2002). Optimization of the MHC class I peptide cargo is dependent on tapasin. Immunity *16*, 509–520.

Wittenbrink, N., Herrmann, S., Blazquez-Navarro, A., Bauer, C., Lindberg, E., Reinke, P., Sawitzki, B., Thomusch, O., Hugo, C., Babel, N., et al. (2019). A novel approach reveals that HLA class 1 single antigen bead-signatures provide a means of high-accuracy pre-transplant risk assessment of acute cellular rejection in renal transplantation. BMC Immunol. *20*, 11.

STAR★METHODS

KEY RESOURCES TABLE

REAGENT or RESOURCE	SOURCE	IDENTIFIER
Antibodies		
Anti-TAPBPR mAb, PeTe4	Boyle et al., 2013	N/A
Anti-TAPBPR mAb	abcam	Cat# ab57411
Anti-tapasin Pasta-1	Gift from Peter Cresswell	N/A
Anti-tapasin R.gp48N	Gift from Peter Cresswell	N/A
HC10	Stam et al., 1986	N/A
HCA2	Stam et al., 1990	N/A
Anti-human β_2m	Dako	Cat# A0072
W6/32	Barnstable et al., 1978	N/A
Anti-calnexin mAb	Enzo Life Sciences	Cat# ADI-SPA-860; RRID: AB_10616095
Mouse IgG2A isotype control	Sigma-Aldrich	Cat# X0943
Goat anti-mouse Alexa Fluor 647 IgG	Invitrogen Molecular Probes, Thermo Fisher Scientific	Cat# A21236; RRID: AB_2535805
Chemicals, Peptides, and Recombinant Proteins		
FuGENE	Promega	Cat# E2311
Hexadimethrine bromide	Sigma-Aldrich	Cat# H9268
ETVSK(5TAMRA)QSNV peptide	Peptide Synthetics	N/A
YLLEK(5TAMRA)LWRL peptide	Peptide Synthetics	N/A
KTGGPIYK(5TAMRA)R peptide	Peptide Synthetics	N/A
PYLFK(5TAMRA)LAAI peptide	Peptide Synthetics	N/A
RVLDK(5TAMRA)VEKW peptide	Peptide Synthetics	N/A
SRYWK(5TAMRA)IRTR peptide	Peptide Synthetics	N/A
SHETK(5TAMRA)IIEL peptide	Peptide Synthetics	N/A
SPAIK(5TAMRA)QSSM peptide	Peptide Synthetics	N/A
EEFGK(5TAMRA)AFSF peptide	Peptide Synthetics	N/A
LNPSK(5TAMRA)AATL peptide	Peptide Synthetics	N/A
Recombinant soluble TAPBPR WT	Ilca et al., 2018b	N/A
Recombinant soluble TAPBPR TN5 mutant	Ilca et al., 2018b	N/A
Critical Commercial Assays		
LABScreen Single Antigen HLA Class I CQ14NC7, Lot 011– HLAF Catalog File	One Lambda, Inc., CA, USA	Cat# LS1A04
Experimental Models: Cell Lines		
HEK293T	Neerincx and Boyle, 2019	N/A
HeLaM-HLA-ABC^{KO}	Neerincx and Boyle, 2019	N/A
Oligonucleotides		
Primers for HLA class I mutants, see Table S3	This paper	N/A
Recombinant DNA		
pHRSINcPPT-SGW	Boyle et al., 2013	N/A
PiggyBac transposon vector	Ilca et al., 2018b	N/A
cDNA of HLA I alleles	Kind gifts from Peter Parham, Elisabeth Chalmeau, Jane Goodall, Ashley Moffett, Sebastian Springer, Rajiv Khanna and Jim McCluskey	N/A
Software and Algorithms		
HLA Fusion^{TM} software	One Lambda, Inc., CA, USA	https://www.onelambda.com/en/product/hla-fusion.html

LEAD CONTACT AND MATERIALS AVAILABILITY

Further information and requests for reagents may be directed to and will be fulfilled by the Lead Contact, Louise H. Boyle (lhb22@cam.ac.uk).

This study did not generate new unique reagents.

EXPERIMENTAL MODEL AND SUBJECT DETAILS

The human cell lines HeLaM-HLA-ABC^{KO}, generated as previously described (Neerincx and Boyle, 2019), and HEK293T were maintained in Dulbecco's Modified Eagle's medium (DMEM; Sigma-Aldrich, UK) supplemented with 10% fetal bovine serum (FBS) (GIBCO, Thermo Fisher Scientific), 100 U/ml penicillin and 100 μg/ml streptomycin (GIBCO, Thermo Fisher Scientific), at 37°C with 5% CO_2.

METHOD DETAILS

Constructs

cDNA templates for a large panel HLA I molecules were kind gifts from Peter Parham (Stanford University), Elisabeth Chalmeau (University Nantes, France), Jane Goodall (University of Cambridge, UK), Ashley Moffett (University of Cambridge, UK), Sebastian Springer (Jacobs University, Bremen, Germany), Rajiv Khanna (Queensland Institute of Medical Research, Australia) and Jim McCluskey (University of Melbourne, Australia). These were amplified and cloned into the lentiviral vector pHRSINcPPT-SGW, as previously described (Boyle et al., 2013). The mutant HLA I constructs A*68:02^{Y116D}, A*02:01^{Y116D} and A*68:01^{D114H} were generated as previously described (Ilca et al., 2018a). The mutant constructs A*02:01^{V12M}, A*02:01^{S105P}, A*68:02^{M12V}, A*68:02^{P105S}, B*44:05^{D114H} and C*01:02^{D114H} were generated by quick-change PCR, using primers shown in Table S3. All HLA I mutants were cloned into the pHRSINcPPT-SGW vector. The luminal domains of TAPBPR WT and TAPBPR TN5 mutant (Hermann et al., 2015) were cloned in a PiggyBac transposon vector, as previously described (Ilca et al., 2018b), to produce a secreted version of TAPBPR, containing a polyHis tag at the C terminus, in a mammalian expression system.

Lentiviral transductions

All cells derived from the HeLaM-HLA-ABC^{KO} line, were transduced using lentivirus as previously described (Neerincx and Boyle, 2019); lentivirus-containing supernatant collected from HEK293T cells was added to HeLaM-HLA-ABC^{KO} cells in the presence of 8 μg/mL Hexadimethrine bromide (Sigma-Aldrich). To induce expression of endogenously-expressed TAPBPR and upregulate the surface expression of MHC I molecules, HeLaM-derived cell lines were treated with 200 U/ml IFN-γ (Peprotech, UK) for 48–72 h.

Antibodies

TAPBPR was detected using either PeTe4, a mouse monoclonal antibody (mAb) specific for the native conformation of TAPBPR, raised against amino acids 22–406 of human TAPBPR (Boyle et al., 2013) that does not cross-react with tapasin (Hermann et al., 2013), or ab57411, a mouse mAb raised against amino acids 23–122 of TAPBPR that is reactive to denatured TAPBPR (Abcam, UK). Tapasin was detected using Pasta-1 (Dick et al., 2002), or with R.gp48N, a rabbit polyclonal antibody specific for tapasin (Sadasivan et al., 1996) (kind gifts from Peter Cresswell, Yale University School of Medicine). MHC I heavy chains were detected using mAb HC10 (Stam et al., 1986) and mAb HCA2 (Stam et al., 1990). β_2m was detected using a rabbit polyclonal antibody (Dako, UK). Cell surface MHC I molecules were detected using W6/32, a pan-MHC I mAb that recognizes a conformational epitope on the α2 domain of MHC I, in a manner dependent on presence of β_2m and peptide (Barnstable et al., 1978). Calnexin was detected via western blot analysis using the rabbit polyclonal ADI-SPA-860 (Enzo Life Sciences, UK). A mouse IgG2a isotype control was also used as a control (Sigma-Aldrich).

MHC I-binding peptides

Peptides specific to individual HLA I allotypes were selected using SYFPEITHI database and/or Immune Epitope Database and Analysis Resource. The following MHC I-specific peptides were used: ETVSK*QSNV (K* represents a lysine labeled with 5-carboxytetramethylrhodaime [TAMRA]), derived from the HLA-A*68:02-specific peptide ETVSEQSNV (Ilca et al., 2018a); YLLEK*LWRL, derived from the HLA-A*02:01-specific peptide YLLEMLWRL (Ilca et al., 2018a); KTGGPIYK*R, derived from the HLA-A*68:01-specific peptide KTGGPIYKR (Ilca et al., 2018a); PYLFK*LAAI, derived from the HLA-A*23:01-specific peptide PYLFWLAAI; RVLDK*VEKW, with predicted high affinity for HLA-A*32:01; SRYWK*IRTR, derived from the HLA-B*27:05-specific peptide SRYWAIRTR; SPAIK*QSSM, derived from the HLA-B35 and -B7-specific peptide SPAIFQSSM; SHETK*IIEL, derived from the HLA-B*38:01-specific peptide *SHETVIIEL*; EEFGK*AFSF, derived from the HLA-B*44:05-specific peptide *EEFGRAFSF*; LNPSK*AATL, derived from the HLA-C*01:02-specific peptide LNPSVAATL. All peptides were purchased from Peptide Synthetics, UK.

Expression and purification of TAPBPR protein

The secreted forms of TAPBPR WT and of TAPBPR TN5 mutant (luminal domain alone), carrying a C-terminal polyHis tag, was expressed in HEK293T cells, using the Piggy Bac expression system, as previously described (Ilca et al., 2018b).

Flow cytometry

Following trypsinization, cells were washed in 1% BSA solution in 1xPBS, at 4°C. Cells were then stained for 30 min, at 4°C, with one of the following antibodies: PeTe4, W6/32 or an isotype control antibody. After two subsequent rounds of washing with 1% BSA solution to remove any excess of unbound antibody, cells were stained for 30 min, at 4°C, with a goat anti-mouse Alexa Fluor 647 IgG (Invitrogen Molecular Probes, Thermo Fisher Scientific). Following another three rounds of washing, the fluorescence levels were detected using a BD FACScan analyzer with Cytek modifications. The analysis was performed using FlowJo (FlowJo, LLC, Ashland, OR).

Peptide binding assays

HeLaM cells were seeded in 12-well plates, at 2.5-3.0*10^4/well, and stimulated with 200 U IFN-γ for 48 h. Cells were then washed with 1x PBS and then incubated in opti-MEM (GIBCO, Thermo Fisher Scientific), at physiological pH, with either 100 nM or 1 μM TAPBPR at 37°C. After 15 min, fluorescently labeled peptide was added to the cells, at different concentrations (10 nM for A*68:02, A*02:01, A*23:01, A*32:01, B*44:05 and 100 nM for A*68:01, A*03:01, A*11:01, B*27:05, B*38:01, C*01:02, C*02:02) and for different time periods (15 min for A*68:02, A*23:01 and 60 min for A*02:01, A*32:01, A*68:01, A*03:01, A*11:01, B*27:05, B*38:01, B*44*05, C*01:02, C*02:02), depending on the HLA I allotype expressed. Cells were then washed three times in 1x PBS to remove any excess of unbound TAPBPR and peptide. After cells were harvested, the level of fluorescent peptide bound was measured by flow cytometry, using the YelFL1 channel (Cytek).

BFA decay assays

IFN-γ-stimulated HeLa-HLA-ABC^{KO} cells, reconstituted with individual HLA I allotypes were treated with 10 μg/mL BFA (Sigma-Aldrich) for different time periods. Cells were then harvested and the levels of MHC class I molecules present at the surface of each cell line, at each time point, were measured by flow cytometry, by staining with the W6/32 antibody.

Single antigen bead screen

3 μL of the LABScreen® single antigen HLA bead suspension (One Lambda, Inc., CA, USA) was added per well of a 96-well plate and incubated with either 100 nM or 1 μM soluble WT TAPBPR, or with 1 μM TAPBPR TN5 mutant, at 22°C, with rotation, for 60 min. The beads were washed three times in wash buffer (One Lambda, Inc., CA, USA) to remove any excess of soluble TAPBPR and were then first incubated with PeTe4 antibody for 30 min, washed and then incubated with a PE-conjugated goat anti-mouse IgG (Abcam, UK) for another 30 min at 22°C. After a subsequent round of washing, cells were re-suspended in 1x PBS and the TAPBPR levels bound to the beads were measured by a using the Luminex Fluoroanalyser system (One Lambda, Inc., CA, USA) and analyzed using the HLA Fusion™ software (One Lambda, Inc., CA, USA).

Immunoprecipitation, gel electrophoresis and western blotting

5x10^6 IFN-γ-stimulated HeLaM-HLA-ABC^{KO} cells reconstituted with each HLA I molecule were harvested and washed in PBS. Cells were then lysed either in 1% Triton X-100 (VWR, Radnor, PN) in Tris-buffered saline (TBS) (20 mM Tris-HCl, 150 mM NaCl, 2.5 mM $CaCl_2$), whenever TAPBPR was pulled down, or in 1% digitonin (Merck, MA, USA) in TBS, whenever tapasin was pulled down, supplemented with 10 mM NEM, 1 mM phenylmethylsulfonyl fluoride (PMSF) (Sigma-Aldrich), and protease inhibitor cocktail (Roche, UK), for 30 min at 4°C. Nuclei and debris were removed by centrifugation at 13,000 x g for 15 min. For the recombinant TAPBPR pull-down experiments, 2 μg of TAPBPR was added per sample and incubated for 1 h at 4°C with rotation, following which TAPBPR was pulled down with beads pre-coated with 5 μg PeTe4 antibody/sample, again for 1 h at 4°C. Endogenous TAPBPR and tapasin were pulled down from the supernatants by using beads coated with either PeTe4 or Pasta-1 antibody, respectively, at 5 μg antibody/sample. After immunoprecipitation, beads were washed thoroughly in 0.1% detergent-TBS to remove unbound protein. The separation by gel electrophoresis was performed as previously described (Ilca et al., 2018b). Samples were then were transferred onto an Immobilon transfer membrane (Merck Millipore) and analyzed by western blotting as previously described (Neerincx et al., 2017).

QUANTIFICATION AND STATISTICAL ANALYSIS

Quantification of protein or peptide binding at the cell surface, measured by flow cytometry was performed using the FlowJo analysis software, version 10. Statistical analysis was performed using GraphPad Prism. Statistical tests and significant values are included in the figure legends.

DATA AND CODE AVAILABILITY

This study did not generate any unique datasets or code.

Cell Reports, Volume *29*

Supplemental Information

Distinct Polymorphisms in HLA Class I Molecules Govern Their Susceptibility to Peptide Editing by TAPBPR

F. Tudor Ilca, Linnea Z. Drexhage, Gemma Brewin, Sarah Peacock, and Louise H. Boyle

Supplementary information

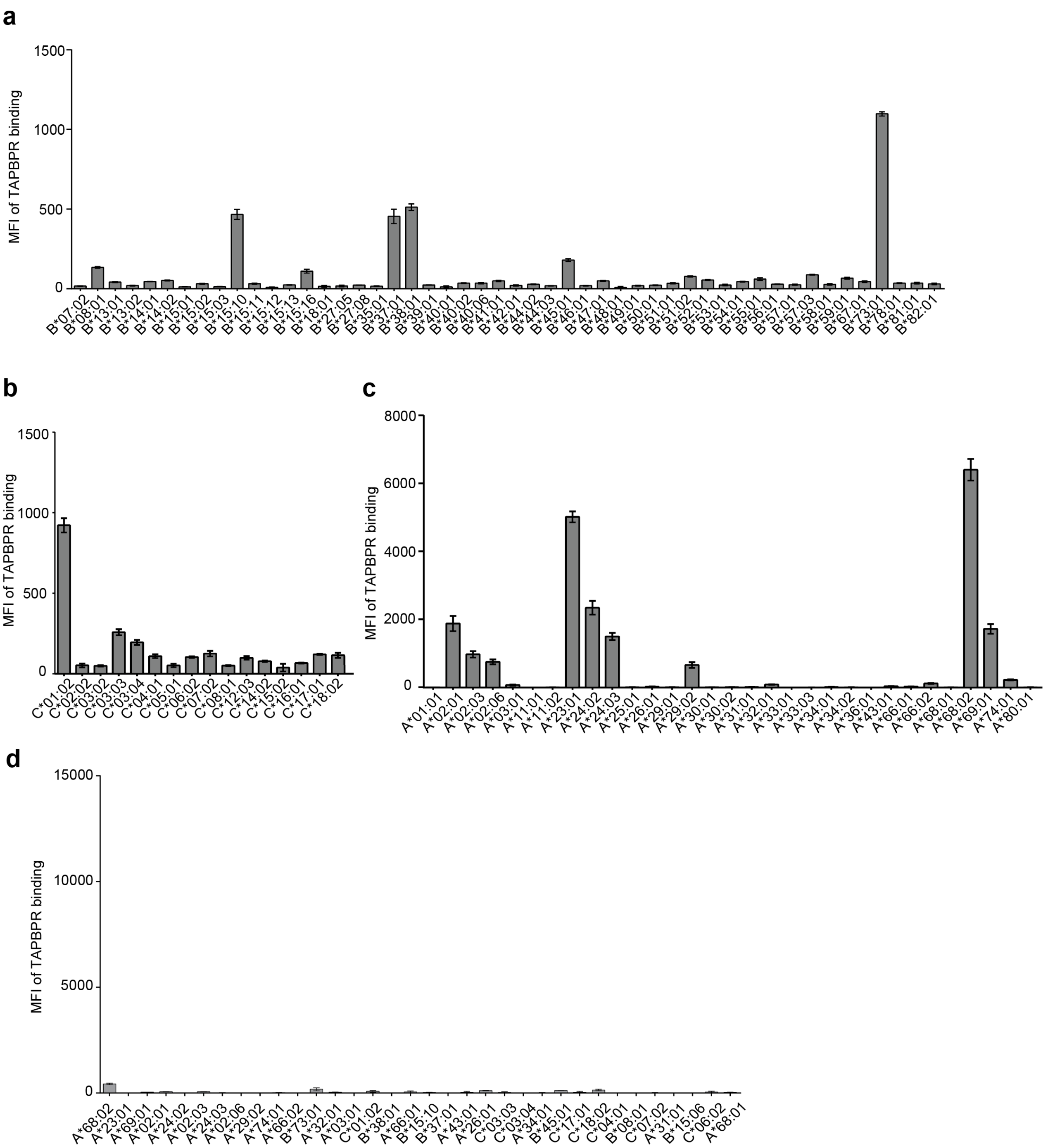

Figure S1. TAPBPR binding to HLA-B and -C molecules on the single HLA beads, Related to Figure 1. Bar graphs showing soluble TAPBPR binding to (**a**) HLA-B and (**b**) HLA-C molecules, upon treatment with 1 μM TAPBPR, to (**c**) HLA-A molecules treated with 100 nM TAPBPR and (**d**) with 1μM TN5 TAPBPR mutant to the top 34 HLA I binders to WT TAPBPR, as shown in **figure 1b**, using the SAB library. The SABs were treated with TAPBPR for 1 h at 22°C. The data including error bars was generated based on triplicates within one experiment. This is a representative example of three independent experiments.

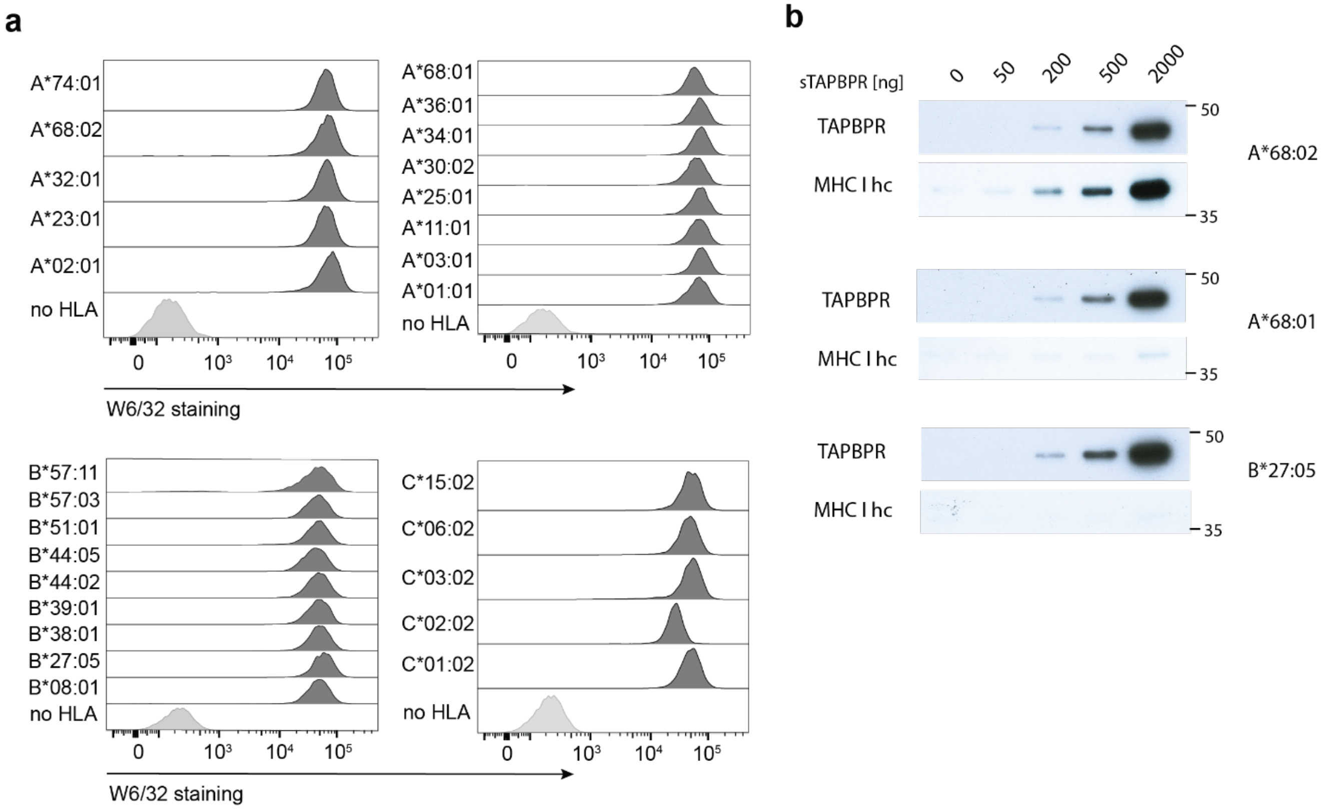

Figure S2. Levels of cell surface MHC class I molecules for individual HLA I alleles, Related to Figure 2 and Figure 3. (**a**) Histograms showing MHC class I levels detected with W6/32 antibody for each HLA I molecule transduced into HeLaM-HLA-ABC^{KO} cells (dark grey filled histograms). A light grey filled histogram was included for non-transduced HeLaM-HLA-ABC^{KO} cells. This is a representative example of three independent experiments. (**b**) Western blot analysis on recombinant TAPBPR pull-downs, when recombinant TAPBPR was titrated, on cells expressing A*68:02, A*68:01 or B*27:05. Membranes were probed for MHC I heavy chain (using HC10) and TAPBPR, as indicated. This is a representative experiment of two independent repeats.

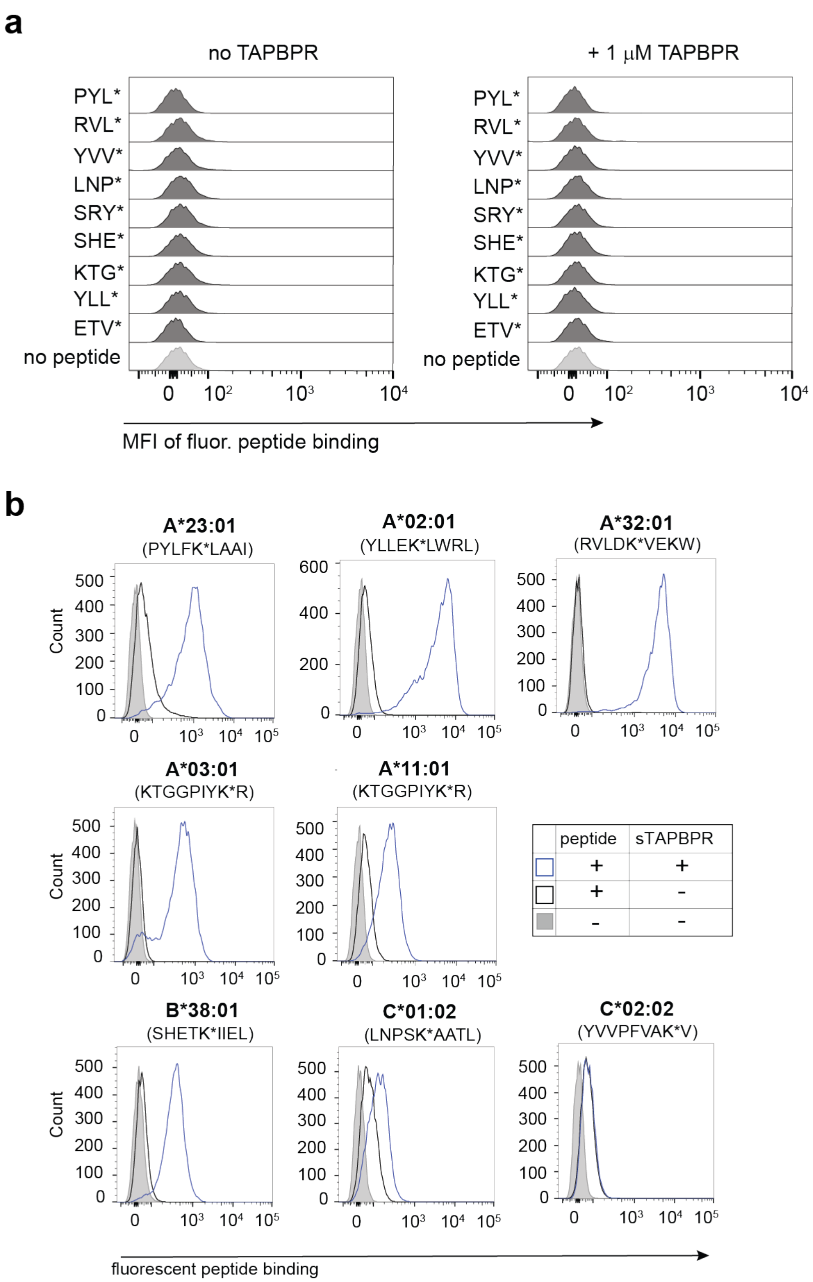

Figure S3. None of the peptides bind to HeLaM cells lacking classical HLA I molecules, Related to Figure 4. (**a**) Histograms showing binding levels of the fluorescent peptides used in **figure 4c** to HeLaM-HLA-ABC^{KO} cells, in the presence (right) or absence (left) of 1 μM TAPBPR. Cells were treated with TAPBPR for 15 min at 37°C, then peptide was added for 1 h at 37°C. The peptides tested were: ETVSK*QSNV (ETV*), YLLEK*LWRL (YLL*), KTGGPIYK*R (KTG*), SHETK*IIEL (SHE*), SRYWK*IRTR (SRY*), LNPSK*AATL (LNP*), YVVPFVAK*V (YVV*), RVLDK*VEKW (RVL*) and PYLFK*LAAI (PYL*). (**b**) Histograms showing the level of fluorescent peptide bound to individual HLA I-expressing cells, additional to the ones depicted in **figure 4b**, either untreated (filled grey line), treated with peptide alone (black line) or with peptide and TAPBPR (blue line). These histograms are representative of three independent experiments.

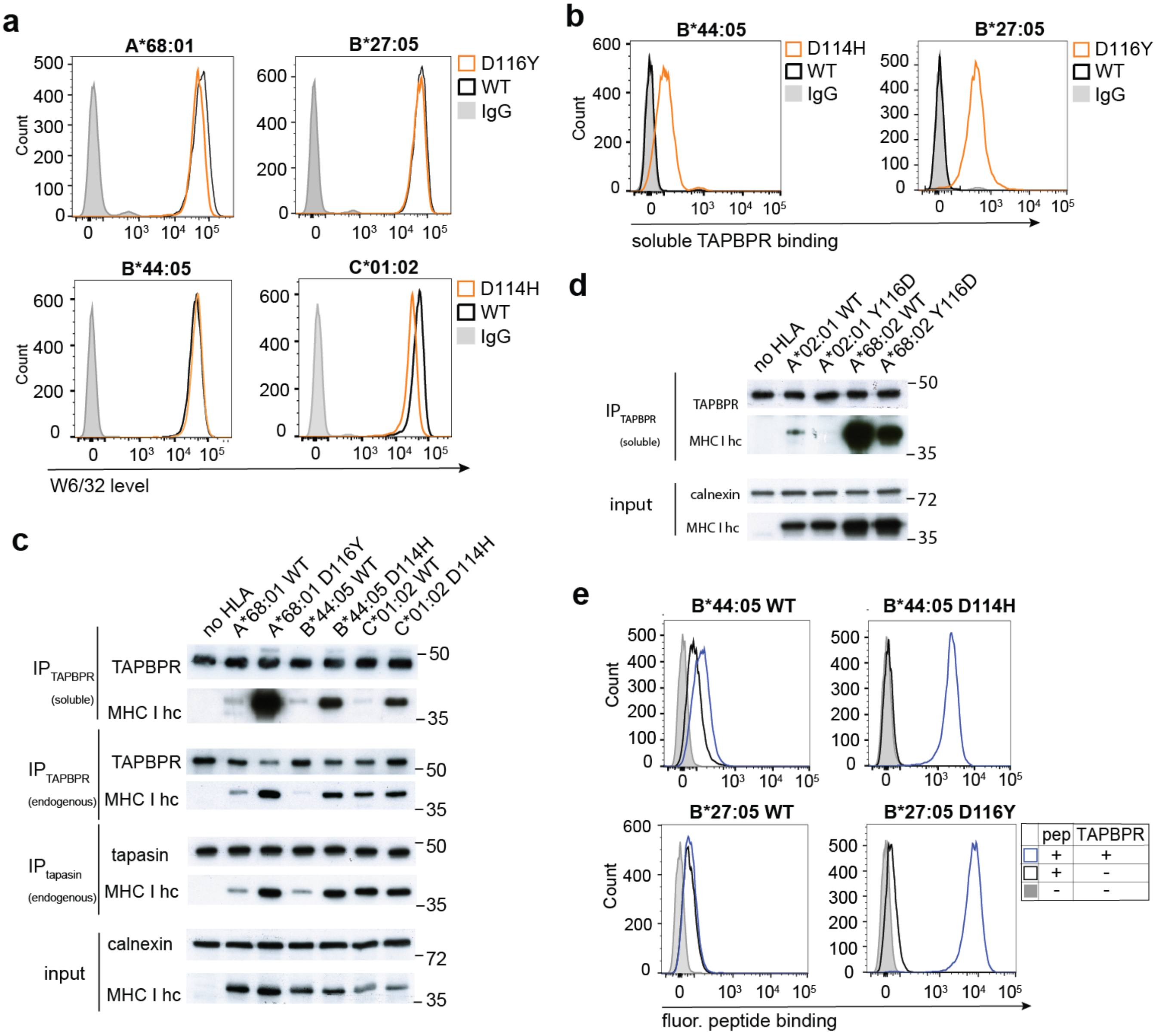

Figure S4. Disrupting the F pocket architecture impairs HLA I binding to TAPBPR, Related to Figure 6. (**a**) Histograms showing surface expression levels of A*68:01, B*27:05, B*44:05 and C*01:02 (black lines), compared to their corresponding F pocket mutants (orange lines). (**b**) Histograms showing the level of bound TAPBPR to cell surface B*44:05 or B*27:05 (black lines), as well as to their corresponding F pocket mutants (orange lines). Untreated cells are included as a negative control (solid grey line). (**c**) Western blot analysis on recombinant TAPBPR pull-downs (top) and endogenous TAPBPR and tapasin immunoprecipitates (bottom), on cells expressing A*68:01^{WT}, A*68:01^{D116Y}, B*44:05^{WT}, B*44:05^{D114H}, C*01:02^{WT} and C*01:02^{D114H} and on (**d**) recombinant TAPBPR pull-downs on cells expressing A*02:01, A*68:02, as well as their Y116D mutants. Membranes were probed for MHC I heavy chain, TAPBPR, tapasin, and calnexin, as indicated. (**e**) Histograms showing the level of bound fluorescent peptides (top) EFGK*AFSF to cells expressing either B*44:05^{WT} or B*44:05^{D114H}, and (bottom) SRYWK*IRTR to cells expressing either B*27:05^{WT} or B*27:05^{D116Y}, when cells were incubated with peptide alone (black line) or with peptide and 1 µM TAPBPR (blue line); an untreated sample was included as a negative control (solid grey line).

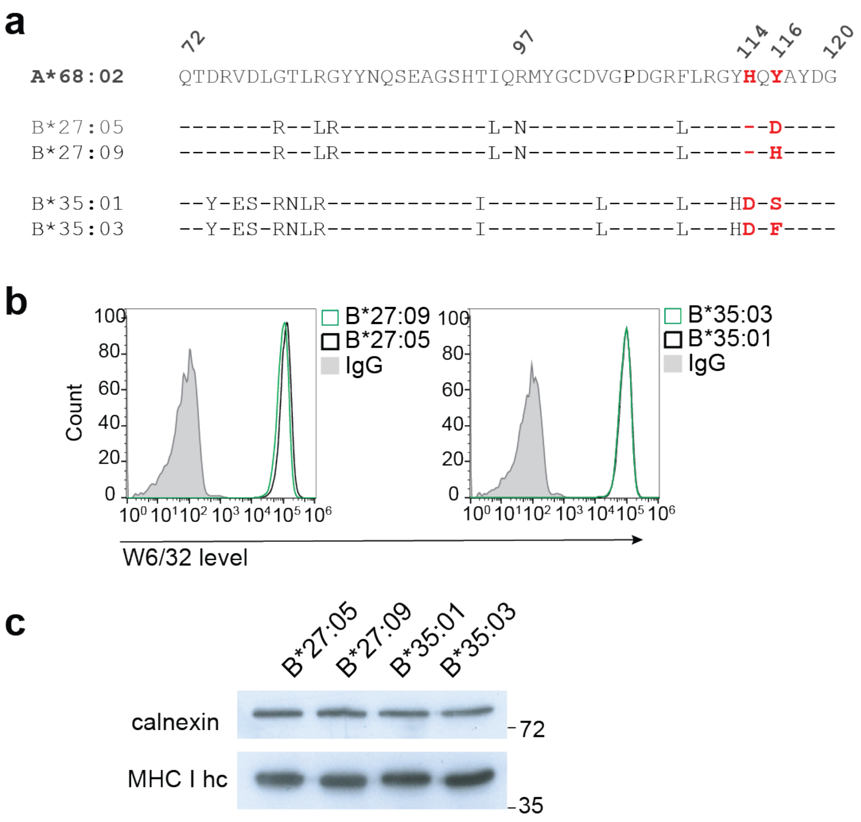

Figure S5. Expression of HLA-B molecules with natural polymorphisms at residue 116, Related to Figure 6. (**a**) Amino acid sequence alignment comparing residues 72-120 of A*68:02 with the HLA I pairs B*27:05 - B*27:09 and B*35:01 - B*35:03; residues 114 and 116 are highlighted in red. (**b**) Histograms showing the surface expression levels of the HLA-B molecules listed in (a), detected with W6/32 antibody. (**c**) Western blot analysis of the expression levels of HLA I molecules in whole cell lysates (a). Membranes were probed for MHC I heavy chain and calnexin, as indicated. These experiments are representative examples of three independent repeats.

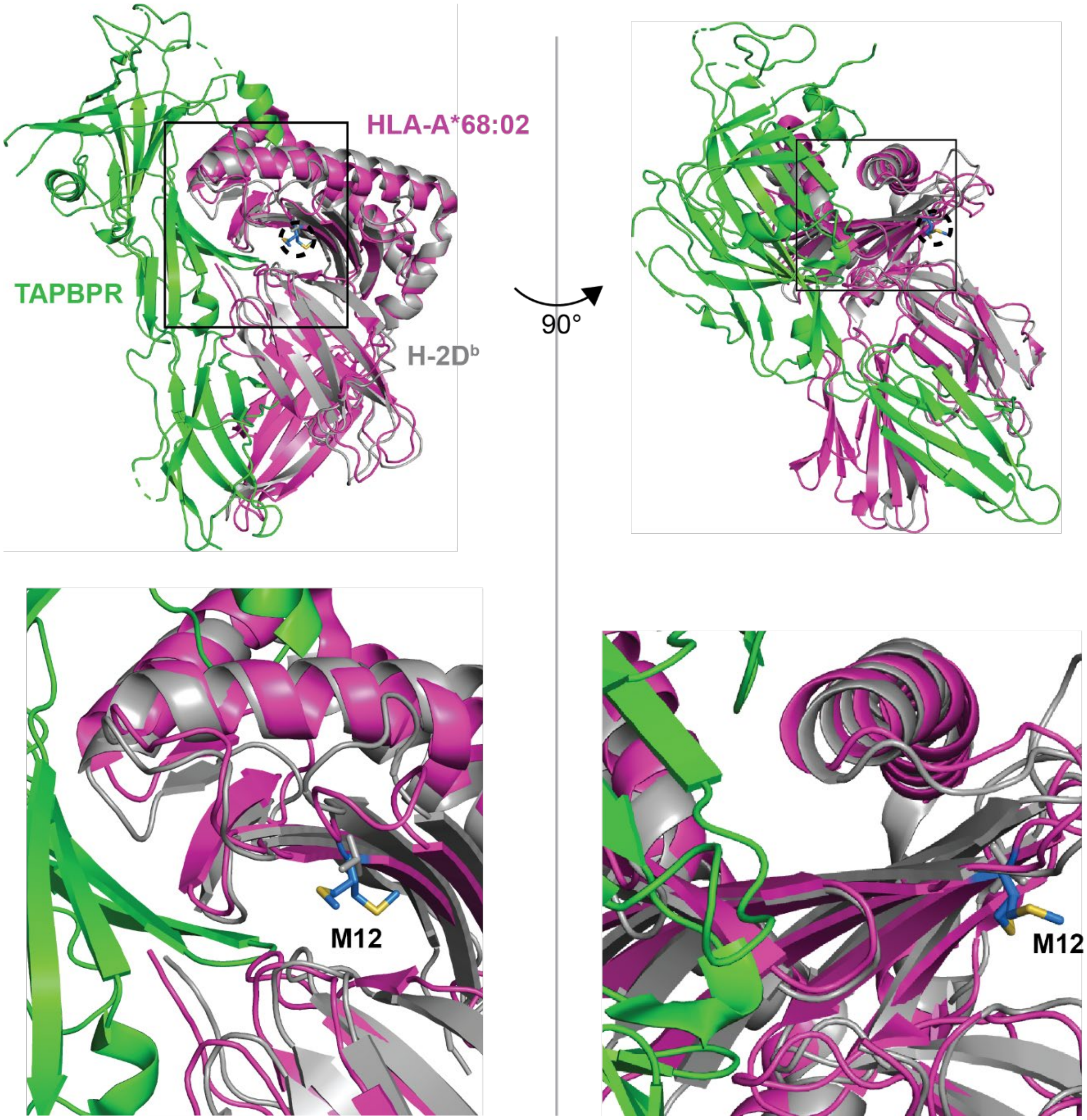

Figure S6. Residue M12 of HLA-A*68:02 does not appear to directly interact with TAPBPR, Related to Figure 7. PyMOL figure of the structure of HLA-A*68:02 folded with peptide SVYDFFVWL (pink) (PDB ID 4HX1) overlaid onto the structure of the H-$2D^b$:TAPBPR complex (grey and green respectively) (PDB ID 5OPI), depicted from different angles. Residue M12 of HLA-A*68:02 is colored in blue and highlighted in a dotted circle. Magnified selected areas are depicted below the whole structures. Residue M12 was captured in two different orientations in the crystal structure of HLA-A*68:02.

Table S1. MHC class I levels on single antigen HLA beads detected using W6/32, Related to Figure 1.

HLA allele	Bead #	MHC class I levels
A*01:01	3	18527
A*02:01	4	19739
A*02:03	5	18974
A*02:06	6	18702
A*03:01	7	19116
A*11:01	8	18910
A*11:02	9	19415
A*23:01	10	19552
A*24:02	11	19068
A*24:03	12	17692
A*25:01	13	19499
A*26:01	14	18366
A*29:01	15	19014
A*29:02	17	18142
A*30:01	18	17818
A*30:02	19	17921
A*31:01	20	18716
A*32:01	21	19548
A*33:01	22	18132
A*33:03	100	18062
A*34:01	23	19320
A*34:02	24	19845
A*36:01	25	17031
A*43:01	26	18328
A*66:01	27	18555
A*66:02	28	19088
A*68:01	29	19016
A*68:02	30	17506
A*69:01	31	18749
A*74:01	32	19216
A*80:01	33	18535
B*07:02	34	20419
B*08:01	35	20346

HLA allele	Bead #	MHC class I levels
B*13:01	97	18064
B*13:02	36	19727
B*14:01	37	19502
B*14:02	38	17233
B*15:01	40	19608
B*15:02	41	19350
B*15:03	42	19668
B*15:10	43	20197
B*15:11	98	17639
B*15:12	44	19294
B*15:13	45	19296
B*15:16	46	18461
B*18:01	47	19915
B*27:05	16	19969
B*27:08	48	19129
B*35:01	49	19066
B*37:01	50	18551
B*38:01	51	18845
B*39:01	52	20136
B*40:01	53	19119
B*40:02	54	19336
B*40:06	99	18638
B*41:01	55	20023
B*42:01	56	20116
B*44:02	57	17659
B*44:03	58	18984
B*45:01	59	19214
B*46:01	63	19173
B*47:01	61	17077
B*48:01	62	19200
B*49:01	60	19506
B*50:01	64	19141
B*51:01	65	19516

HLA allele	Bead #	MHC class I levels
B*51:02	66	18795
B*52:01	67	17497
B*53:01	68	18690
B*54:01	69	19465
B*55:01	70	19803
B*56:01	71	19119
B*57:01	72	18956
B*57:03	73	19594
B*58:01	74	18524
B*59:01	75	18090
B*67:01	76	19989
B*73:01	77	19542
B*78:01	78	17716
B*81:01	79	18679
B*82:01	80	17708
C*01:02	81	18988
C*02:02	82	16840
C*03:02	83	18982
C*03:03	84	19000
C*03:04	85	18943
C*04:01	86	12491
C*05:01	87	19122
C*06:02	88	16573
C*07:02	89	18133
C*08:01	90	19692
C*12:03	91	20447
C*14:02	92	17786
C*15:02	93	21276
C*16:01	94	14783
C*17:01	95	14369
C*18:02	96	18683

Table S2. Characterization of HLA-A molecules subjected to TAPBPR binding using the single antigen HLA beads, Related to Figure 5.

HLA allomorph	Supertype	F pocket specificity	Residue 114	Residue 116
**A*68:02**	**A2**	**Hydrophobic**	H	Y
**A*23:01**	**A24**	**Hydrophobic**	H	Y
**A*69:01**	**A2**	**Hydrophobic**	H	Y
**A*02:01**	**A2**	**Hydrophobic**	H	Y
**A*24:02**	**A24**	**Hydrophobic**	H	Y
**A*02:06**	**A2**	**Hydrophobic**	H	Y
A*03:01	A3	Basic	R	D
A*01:01	A1	Aromatic	R	D
A*26:01	A1	Aromatic	Q	D
A*30:01	A3	Basic / Aromatic	E	H
A*30:02	A1	Aromatic	E	H
A*33:01	A3	Basic	Q	D
A*36:01	A3	Basic	R	D
A*66:01	A3	Basic	Q	D
A*68:01	A3	Basic	R	D

- strong TAPBPR binders are highlighted in bold

Table S3: Primers used for cloning the MHC class I mutants, Related to STAR Methods.

Primer name	Sequence 5'-3'
B2705_D116Y_Fwd	GTACCACCAGTACGCCTACG
B2705_D116Y_Rev	CGTAGGCGTACTGGTGGTAC
B4405_D114H_Fwd	CGCGGGTATCATCAGTACGC
B4405_D114H_Rev	GCGTACTGATGATACCCGCG
C0102_D114H_Fwd	GCGGGTATCACCAGTACGC
C0102_D114H_Rev	GCGTACTGGTGATACCCGC
A0201_V12M_Fwd	CACATCCATGTCCCGGCC
A0201_V12M_Rev	GGCCGGGACATGGATGTG
A0201_S105P_Fwd	GACGTGGGGCCGGACTGG
A0201_S105P_Rev	CCAGTCCGGCCCCACGTC
A6802_M12V_Fwd	CTACACTTCCGTGTCCCGGC
A6802_M12V_Rev	GCCGGGACACGGAAGTGTAG
A6802_P105S_Fwd	CGTGGGGTCGGACGGG
A6802_P105S_Rev	CCCGTCCGACCCCACG
